# Antidiabetic Effects of *Ecklonia cava* and Dieckol via DPP-IV Inhibition and Glucose Transport Regulation

**DOI:** 10.3390/md24050174

**Published:** 2026-05-12

**Authors:** Indyaswan T. Suryaningtyas, Nabila Shafura, Ratih Pangestuti, Won-Kyo Jung, Jae-Young Je

**Affiliations:** 1 Research Center for Marine-Integrated Bionics Technology, Pukyong National University, Busan 48513, Republic of Korea; indy001@brin.go.id (I.T.S.); wkjung@pknu.ac.kr (W.-K.J.); 2 Research Center for Food Technology and Processing, National Research and Innovation Agency (BRIN), Yogyakarta 55861, Indonesia; rati008@brin.go.id; 3 Major of Human Bioconvergence, Division of Smart Healthcare, Pukyong National University, Busan 48513, Republic of Korea; nabila@pukyong.ac.kr; 4 Marine Integrated Biomedical Technology Center, The National Key Research Institutes in Universities, Pukyong National University, Busan 48513, Republic of Korea; 5 Major of Biomedical Engineering, Division of Smart Healthcare, College of Information Technology and Convergence and New-Senior Healthcare Innovation Center (BK21 Plus), Pukyong National University, Busan 48513, Republic of Korea

**Keywords:** *Ecklonia cava*, dieckol, DPP-IV, glucose transporters, AMPK, type 2 diabetes, phlorotannins

## Abstract

Brown seaweeds are recognized for their rich content of phlorotannins with promising antidiabetic properties through multi-targeted modulation of glucose metabolism. This study investigated the antidiabetic potential of the ethyl acetate fraction of *Ecklonia cava* (EC-ETAC) and its major phlorotannin, dieckol, focusing on inhibition of carbohydrate-digesting enzymes, intestinal glucose absorption, dipeptidyl peptidase-IV (DPP-IV) activity, and hepatic glucose metabolism. EC-ETAC potently inhibited α-glucosidase (IC_50_ = 2.2 ± 0.2 µg/mL) and α-amylase (IC_50_ = 41.0 ± 1.2 µg/mL), outperforming acarbose by 26-fold and 6-fold, respectively. Pure dieckol showed strong activity with IC_50_ values of 2.213 ± 0.04 µM (α-glucosidase) and 156.87 ± 0.124 µM (α-amylase). In differentiated Caco-2 cells, both EC-ETAC and dieckol downregulated SGLT1 and GLUT2 protein expression to ~0.5-fold of control and suppressed 2-NBDG glucose uptake by 46–53% over 120 min, effects not seen with acarbose. Dieckol inhibited DPP-IV activity (IC_50_ = 12.12 ± 0.021 µM), reducing in situ activity to 53.89% at 25 µM without changing DPP-IV protein levels. Molecular docking revealed high-affinity binding of dieckol to DPP-IV (−10.396 kcal/mol), directly occluding the catalytic triad (Ser630, His740). In insulin-resistant HepG2 cells, dieckol restored glucose uptake to 108.97% of control via AMPK activation (1.21-fold), GLUT2 normalization (0.84-fold), and PGC-1α recalibration (0.96-fold), matching or surpassing 1 mM metformin. These results demonstrate dual-inhibition mechanism combined with hepatic AMPK restoration, establishing dieckol as a promising marine-derived multi-targeted agent for T2DM management.

## 1. Introduction

Type 2 diabetes mellitus (T2DM) represents a rapidly escalating global health burden [1,2]. The disease is characterized by postprandial hyperglycemia resulting from impaired carbohydrate digestion and absorption, rapid degradation of incretin hormones such as glucagon-like peptide-1 (GLP-1), and hepatic insulin resistance that disrupts glucose uptake and promotes excessive gluconeogenesis [3,4,5]. Current pharmacological strategies frequently exhibit gastrointestinal side effects, limited long-term efficacy, high cost, and incomplete coverage of multi-organ metabolic dysfunction, necessitating safer, multi-targeted alternatives from natural sources [6,7].

Among the key therapeutic targets in T2DM management is dipeptidyl peptidase-IV (DPP-IV), a serine protease widely expressed on the brush-border membrane of intestinal epithelial cells, vascular endothelium, and hepatocytes [8,9]. DPP-IV rapidly cleaves and inactivates incretin hormones, particularly GLP-1, thereby diminishing glucose-dependent insulin secretion, accelerating gastric emptying, and reducing satiety [10]. Inhibition of DPP-IV prolongs the half-life of active GLP-1, leading to enhanced insulin release, suppressed glucagon secretion, and improved postprandial glycemic control [8,9]. Synthetic DPP-IV inhibitors such as gliptins are clinically effective; however, concerns regarding long-term safety, cost, and potential off-target effects have driven interest in natural alternatives [11,12,13]. Phlorotannin-rich extracts from brown seaweeds have shown preliminary DPP-IV inhibitory activity in cell-free systems, suggesting that compounds may serve as effective natural modulators of the incretin axis by directly interacting with the enzyme’s catalytic site [14,15,16,17].

Unlike terrestrial polyphenols, phlorotannins possess unique dibenzo-1,4-dioxin linkages and high hydroxyl density that confer strong enzyme-binding capacity [18,19,20]. *Ecklonia cava* (EC) has emerged as a key species, with its ethyl acetate fractions and isolated dieckol demonstrating potent inhibition of α-glucosidase and α-amylase in vitro and significant reductions in postprandial glucose excursion in streptozotocin-induced diabetic mice [21,22,23]. In db/db mouse models, dieckol administration improved hyperglycemia and insulin sensitivity through activation of AMP-activated protein kinase (AMPK) and modulation of lipid metabolism [22,24]. Human trials further support these findings: a single 600 mg dose of EC extract significantly lowered postprandial blood glucose in prediabetic individuals without adverse effects [25]. A comprehensive meta-analysis of 23 randomized controlled trials confirmed that brown seaweed supplementation (>1000 mg/day) consistently reduces postprandial glucose, HbA1c, and HOMA-IR levels, particularly with species such as *Laminaria digitata*, *Undaria pinnatifida*, and *Ecklonia* spp. [26,27]. Broader screening of brown algal phlorotannins has also revealed DPP-IV inhibitory potential in enzymatic experiment, suggesting preservation of incretin function [28,29,30].

Nevertheless, most previous studies on EC and dieckol have primarily examined glucose enzyme inhibition or overall glucose-lowering effects in animal models. Detailed investigations into the modulation of intestinal glucose transporters at the protein level, the direct functional inhibition of DPP-IV within intestinal epithelial cells, and the restoration of hepatic glucose homeostasis through specific signaling pathways in insulin-resistant hepatocytes remain limited. In addition, structural evidence for the molecular interaction between dieckol and the DPP-IV active site has not been reported. The present study therefore evaluated the multi-compartmental antidiabetic actions of the ethyl acetate extract of *Ecklonia cava* (EC-ETAC) and its predominant phlorotannin, dieckol, through integrated enzymatic, intestinal epithelial, and hepatic insulin-resistant models, incorporating molecular docking analysis to elucidate the structural basis of DPP-IV inhibition.

## 2. Result

### 2.1. Inhibitory Effects of EC Various Extracts on α-Glucosidase, α-Amylase, and DPP-IV

The antidiabetic potential of various EC extracts was evaluated by determining their inhibitory activities against key metabolic enzymes. The IC_50_ values for α-glucosidase, α-amylase, and DPP-IV are summarized in Table 1. Among the tested fractions, the ethyl acetate fraction extract (EC-ETAC) exhibited the most potent inhibitory activity across all three enzymes. For α-glucosidase, the EC-ETAC extract recorded the lowest IC_50_ value (2.2 ± 0.2 µg/mL), making it approximately 26 times more potent than the commercial inhibitor, acarbose (57.2 ± 13.0 µg/mL). The n-butanol and ethanol extract also showed significant inhibitory activity, whereas the water extract displayed the lowest potency (121.4 ± 0.8 µg/mL). Regarding α-amylase, the EC-ETAC extract again demonstrated the highest inhibitory effect with an IC_50_ of 41.0 ± 1.2 µg/mL, significantly surpassing the inhibitory capacity of acarbose (250.6 ± 40.2 µg/mL). Meanwhile, the n-butanol and water extracts showed either weak or no detectable inhibitory activity against this enzyme within the tested concentration range.

In the DPP-IV inhibition assay, only the ethanol and EC-ETAC extracts showed measurable activity. The EC-ETAC extract was the most effective fraction (21.0 ± 1.4 µg/mL), although its potency remained lower than that of the reference standard, Diprotin A (6.7 ± 0.12 µg/mL). Based on these screening results, the EC-ETAC fraction was selected as the lead candidate for phytochemical characterization and subsequent mechanistic studies.

### 2.2. Phytochemical Profiling of EC-ETAC via UPLC–Q-TOF-MS/MS

The phytochemical composition of the EC-ETAC fraction was characterized using UPLC–Q-TOF-MS/MS in positive ion mode. Among the complete list of identified compounds, ten major phlorotannins were selected for detailed analysis (Table 2). Identification was confirmed based on high-resolution MS accurate mass (<2 ppm error), [M+H]^+^ adduct formation, and comparison with published literature on EC phlorotannins. All mass errors were maintained within ±2 ppm, confirming high-confidence assignments.

**Figure 1:**
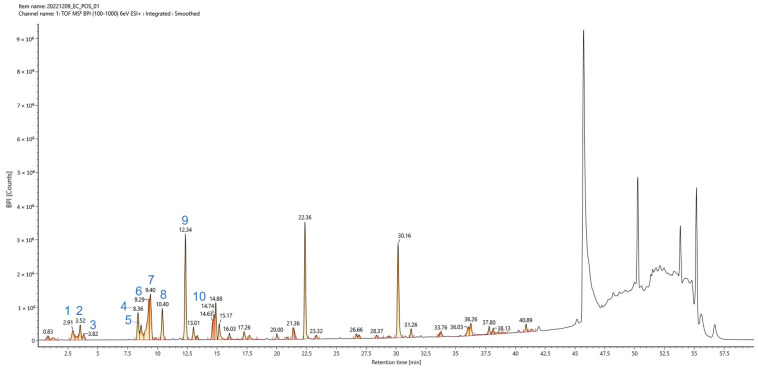
Total ion current (TIC) chromatogram of the EC-ETAC fraction obtained by UPLC–Q-TOF-MS/MS in positive ion mode. The chromatographic separation was performed on a Waters BEH C18 column (2.1 × 100 mm, 1.7 µm) at 40 °C with a gradient elution of water and acetonitrile (both containing 0.1% formic acid) at a flow rate of 0.3 mL/min. Labeled peaks correspond to the ten major phlorotannins listed in Table 2. Unlabeled late-eluting peaks (e.g., at RT 22.36 min and RT 30.16 min) were identified as background contaminants (plasticizers) based on their mass spectra and were excluded as they did not match any compounds in our phlorotannin library.

The total ion chromatogram (TIC) of EC-ETAC is shown in Figure 1, with labeled peaks corresponding to the ten major phlorotannins listed in Table 2. The early-eluting region contained two isomeric forms of fucodiphlorethol G (C_24_H_18_O_12_; [M + H]^+^ *m*/*z* 499.0881 and 499.0878) at 2.90 min and 3.52 min, respectively. The compound at 2.90 min corresponds to the isomer with the systematic name 4′-[2,4-dihydroxy-6-(2,4,6-trihydroxyphenoxy) phenoxy]-2,2′,4,6,6′-biphenylpentol, which differs from fucodiphlorethol G (6′-substituted isomer) in the position of the substituent on the biphenyl core. This was followed by fucophlorethol A (peak 3; C_18_H_14_O_9_; *m*/*z* 375.0717) at 3.83 min. The mid-eluting region contained eckol (peak 4; C_18_H_12_O_9_; *m*/*z* 373.0559) at 8.36 min, 7-phloroeckol (peak 5; C_24_H_16_O_13_; *m*/*z* 497.0721) at 8.62 min, 8,8′-bieckol (peak 6; C_36_H_22_O_18_; *m*/*z* 743.0879) at 9.37 min, and 2-O-(2,4,6-trihydroxyphenyl)-6,6′-bieckol (peak 7; C_42_H_26_O_21_; *m*/*z* 867.1042) at 9.48 min. The later-eluting region contained dibenzodioxin-fucodiphloroethol (peak 8; C_36_H_24_O_19_; *m*/*z* 745.1036) at 10.41 min, followed by dieckol (peak 9; C_36_H_22_O_18_; *m*/*z* 743.0877) as the most intense peak at 12.34 min, and phlorofucofuroeckol A (peak 10; C_30_H_18_O_15_; *m*/*z* 603.0768) at 14.62 min. The extract contained complex high-molecular-weight phlorotannins, with 2-O-(2,4,6-trihydroxyphenyl)-6,6′-bieckol (peak 6; C_42_H_26_O_21_; *m*/*z* 867.1042) exhibiting the highest molecular weight among the identified compounds (867 Da). Notably, two isomeric forms of fucodiphlorethol G (C_24_H_18_O_12_) were detected at RT 2.90 min (peak 1) and 3.52 min (peak 2). Additionally, dieckol and 8,8′-bieckol (peaks 9 and 7) share the same molecular formula C_36_H_22_O_18_ but were clearly separated chromatographically (RT 12.34 vs. 9.37 min), confirming they are positional isomers.

To quantify the major bioactive constituent, the absolute content of dieckol in the EC-ETAC extract was determined by HPLC–DAD using a five-point calibration curve (1–50 mg/L). The calibration curve showed excellent linearity (y = 0.408x + 0.982, R^2^ = 0.993). Quantitative analysis revealed that dieckol comprised 36.29 ± 1.65 mg per gram of the EC-ETAC fraction, equivalent to 3.63% (*w*/*w*) of the total EC-ETAC weight (Supplementary Table S2). This represents a substantial enrichment compared to raw *E. cava* tissue (1.82 mg/g) and falls within the typical range reported for commercial *Ecklonia cava* extracts [31,32,33]. Owing to its high relative abundance and well-documented bioactivity, dieckol was selected as the primary bioactive marker for subsequent mechanistic investigations.

### 2.3. Inhibitory Effects of Dieckol on α-Glucosidase, α-Amylase, and DPP-IV Activities

**Figure 2:**
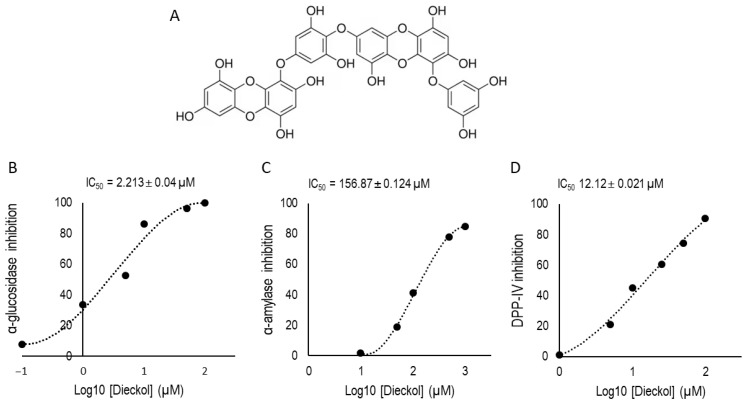
Inhibitory effects of dieckol on metabolic enzymes associated with type 2 diabetes. (**A**) Chemical structure of dieckol, the primary phlorotannin identified in the EC-ETAC fraction. Dose-dependent inhibitory activities of dieckol against (**B**) α-glucosidase, (**C**) α-amylase, and (**D**) DPP-IV. Results are expressed as the mean ± SD of three independent experiments (*n* = 3). IC_50_ values represent the concentration required to inhibit 50% of the respective enzyme activity.

To validate the bioactive potential of the primary phlorotannin identified in the EC-ETAC fraction, the inhibitory effects of pure dieckol (Figure 2A) were evaluated against the three target metabolic enzymes. Dieckol exhibited potent, dose-dependent inhibitory activity in all assays (Figure 2B–D). In the α-glucosidase inhibition assay (Figure 2B), dieckol demonstrated remarkable potency with an IC_50_ of 2.213 ± 0.04 µM. High levels of inhibition were maintained even at lower concentrations, indicating its high affinity for this enzyme. For α-amylase (Figure 2C), the IC_50_ was determined to be 156.87 ± 0.124 µM. Although the concentration required for amylase inhibition was higher than that for glucosidase, dieckol still achieved nearly 90% inhibition at the 1000 µM dosage. Furthermore, dieckol was confirmed as a strong inhibitor of DPP-IV (Figure 2D), yielding an IC_50_ of 12.12 ± 0.021 µM, comparable to well-known DPP-IV inhibitor, Diprotin A with IC_50_ 6.7 ± 0.12 µM.

### 2.4. Molecular Docking Analysis of Dieckol with DPP-IV

**Figure 3:**
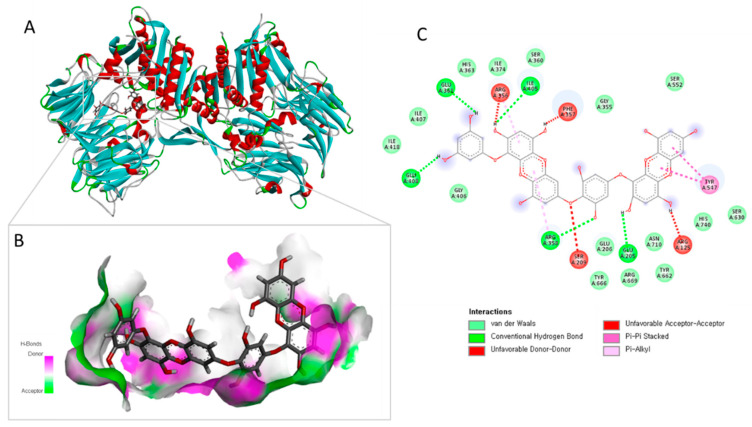
Molecular docking simulation of dieckol with the human DPP-IV receptor. (**A**) 3D ribbon representation of the DPP-IV homodimer. Secondary structures are color-coded: alpha helices (red), beta sheets (cyan), and loops (green). Dieckol (grey sticks) is bound in the deep catalytic pocket of the A-chain. (**B**) Magnified view of the best-scoring pose (Cluster 001; binding energy: −10.396 kcal/mol). (**C**) Detailed 2D ligand-protein interaction map showing the extensive network of chemical bonds. Conventional hydrogen bonds and salt bridges are formed with Arg125, Glu205, Glu206, and Arg358, while hydrophobic and π-stacking interactions occur within the S1 and S2 subpockets involving Phe357, Tyr547, Tyr662, and Tyr666.

To elucidate the molecular basis of the observed DPP-IV inhibitory activity, molecular docking simulations were performed using the crystal structure of human DPP-IV (PDB ID: 1WCY) (Figure 3). Seven independent docking runs yielded binding energies ranging from −9.835 to −10.396 kcal/mol. The top-ranked pose (Cluster 001) displayed the highest binding affinity, with a binding energy of −10.396 kcal/mol and a predicted dissociation constant (Kd) of 23.97 nM. Notably, even the weakest cluster retained a strong binding energy of −9.835 kcal/mol, demonstrating consistent and stable binding poses.

Detailed interaction analysis of the best-scoring pose showed that dieckol binds deeply into the catalytic pocket of DPP-IV. Dieckol directly interacts with the catalytic triad residues Ser630 and His740, thereby blocking the enzyme’s active site. It also forms extensive hydrophobic and π-stacking interactions with the S1 and S2 subpocket residues Tyr547, Tyr662, and Tyr666. The binding is further stabilized by multiple hydrogen bonds and salt bridges with Arg125, Glu205, Glu206, and Arg358 (Figure 3B). These multi-point interactions within the active site provide a clear structural explanation for the potent DPP-IV inhibition observed in both cell-free enzymatic assays and in situ Caco-2 cell experiments, despite no change in total DPP-IV protein expression. The high binding affinity and direct occlusion of the catalytic triad support dieckol as a strong competitive inhibitor of DPP-IV.

### 2.5. EC-ETAC and Dieckol Attenuate Intestinal Glucose Absorption by Inhibiting α-Glucosidase and Downregulating SGLT1/GLUT2 Transporters

**Figure 4:**
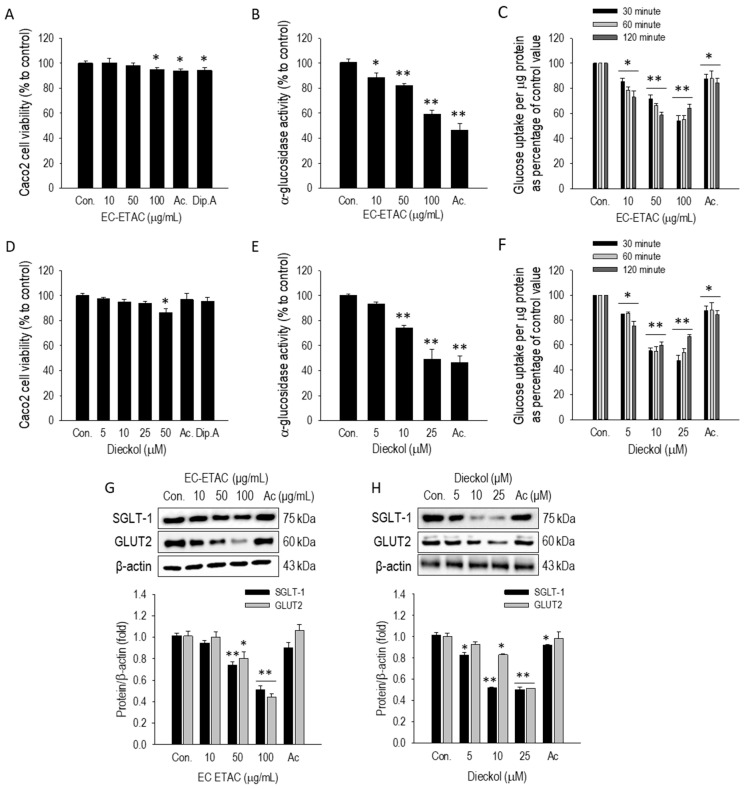
Effects of EC-ETAC and dieckol on intestinal glucose digestion and absorption in Caco-2 cells. (**A**,**D**) Cell viability of differentiated Caco-2 cells, determined via MTT assay after 24 h treatment with varying concentrations of EC-ETAC (10–100 µg/mL) and dieckol (5–50 µM). (**B**,**E**) Inhibitory effects on membrane-bound α-glucosidase activity, measured by the spectrophotometric quantification of *p*-nitrophenol released from the *p*NPG substrate. (**C**,**F**) Time-course (30, 60, and 120 min) of 2-NBDG glucose uptake suppression, assessed by monitoring the intracellular accumulation of the fluorescent glucose analog in 21-day differentiated monolayers. (**G**,**H**) Western blot quantification of protein expression levels for the glucose transporters SGLT1 and GLUT2 from total cell lysates. Acarbose (Ac.) and Diprotin A (Dip. A) were included as reference inhibitors at a final concentration of 100 µM (64.5 µg/mL), and 20 µM (6.7 µg/mL), respectively. Results are expressed as mean ± SD (*n* = 3). Statistical significance compared to the untreated control is indicated by * (*p* < 0.05) and ** (*p* < 0.001) as determined by one-way ANOVA followed by Dunnett’s post hoc test.

To evaluate the safety and functional efficacy of the extract and its lead compound in an intestinal model, Caco-2 cells were treated with varying concentrations of EC-ETAC (10–100 µg/mL) and dieckol (5–50 µM). As shown in Figure 4A,D, cell viability remained above 86% across all tested concentrations for both EC-ETAC and dieckol, indicating no significant cytotoxicity compared to the untreated control. Specifically, at the highest concentration of 100 µg/mL, EC-ETAC maintained a viability of 94.66 ± 2.06%, while 25 µM of dieckol resulted in 86.62 ± 2.87% viability. These concentrations were therefore deemed safe for investigating enzymatic inhibition and transport mechanisms within the cellular environment.

The inhibitory effect on membrane-bound α-glucosidase was further examined using differentiated Caco-2 monolayers. EC-ETAC exhibited a dose-dependent reduction in enzyme activity (Figure 4B), decreasing activity to 59.27 ± 2.84% at 100 µg/mL. Notably, dieckol demonstrated even more potent cellular inhibition (Figure 4E); at 25 µM, dieckol reduced α-glucosidase activity to 49.19 ± 8.08%, which was statistically comparable to the clinical reference, acarbose (46.48 ± 5.06%) at 100 µM concentration (64.56 µg/mL) (Figure 4E). These results suggest that dieckol effectively inhibits the final step of carbohydrate digestion at the intestinal brush border without compromising cell integrity.

Beyond enzymatic inhibition, the effect of EC-ETAC and dieckol on the physical transport of glucose across the intestinal epithelium was evaluated using a 2-NBDG uptake assay over a 120 min period (Figure 4C,F). As shown in Figure 4C, EC-ETAC significantly reduced glucose uptake in a dose- and time-dependent manner; at the 30 min mark, 100 µg/mL of EC-ETAC suppressed uptake to 54.08 ± 3.96%, an effect sustained through 120 min (64.26 ± 3.01%). Dieckol exhibited even more pronounced inhibitory activity, where 25 µM reduced glucose uptake to 47.60 ± 4.04% within 30 min, maintaining potent suppression (53.98 ± 3.21%) at 60 min. Interestingly, the clinical inhibitor acarbose showed minimal impact on glucose transport, with uptake levels remaining high (84.22 ± 3.47% at 120 min).

To elucidate the molecular mechanism underlying this inhibition, the protein expression levels of the primary intestinal glucose transporters, SGLT1 and GLUT2, were analyzed via Western blot (Figure 4G,H). Both EC-ETAC and dieckol significantly suppressed the expression of these transporters in a dose-dependent manner. Treatment with EC-ETAC at 100 µg/mL reduced SGLT1 and GLUT2 expression to 0.51 ± 0.04 and 0.44 ± 0.03 relative to the control, respectively. Similarly, dieckol at 25 µM downregulated SGLT1 to 0.50 ± 0.02 and GLUT2 to 0.51 ± 0.00. In contrast, acarbose failed to induce any significant change in the expression of either transporter (SGLT1: 0.90 ± 0.05; GLUT2: 1.06 ± 0.06). These findings demonstrate that unlike traditional α-glucosidase inhibitors, EC-ETAC and dieckol exert a multi-targeted antidiabetic effect by both inhibiting starch-digesting enzymes and downregulating the key proteins responsible for intestinal glucose absorption.

### 2.6. In Situ Inhibition of DPP-IV Activity and Protein Expression Analysis After EC-ETAC and Dieckol in Caco-2 Cells

**Figure 5:**
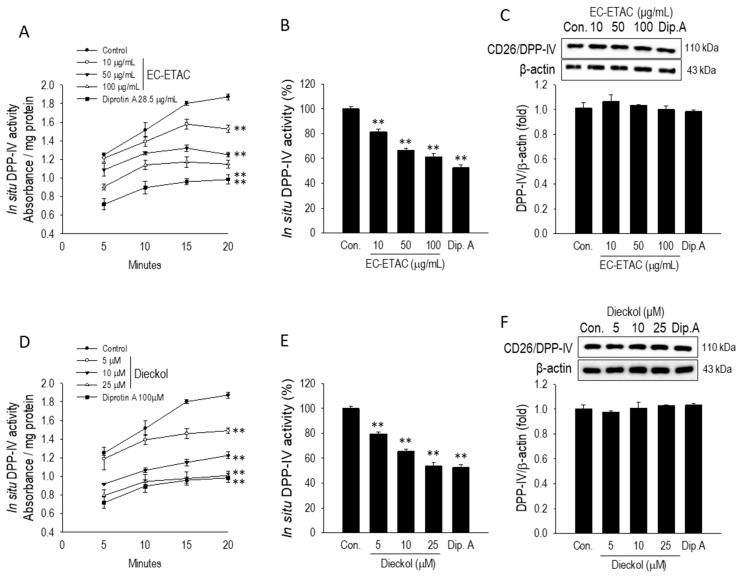
In situ DPP-IV inhibitory kinetics and protein expression in differentiated Caco-2 cells. (**A**,**D**) Time-course (5–20 min) of in situ DPP-IV absorbance per mg protein following treatment with EC-ETAC (10–100 µg/mL) and dieckol (5–25 µM), respectively. (**B**,**E**) Percentage of in situ DPP-IV activity relative to the untreated control at the 20 min endpoint, assessed by the spectrophotometric liberation of *p*-nitroaniline from the Gly-Pro-*p*-nitroanilide substrate. (**C**,**F**) Western blot representative images and quantification of DPP-IV protein expression, demonstrating no significant difference across treatment groups. 20 µM or 6.7 µg/mL Diprotin A (Dip. A) served as the positive reference. Results are expressed as mean ± SD (*n* = 3). Statistical significance compared to the untreated control is indicated by ** (*p* < 0.001) as determined by one-way ANOVA with Dunnett’s post hoc test.

To bridge the gap between cell-free enzyme assays and functional cellular environments, the inhibitory effects of EC-ETAC and dieckol on in situ DPP-IV activity were monitored over a 20 min kinetic period in differentiated Caco-2 cells. As illustrated in the absorbance kinetics (Figure 5A,D), both treatments suppressed the rate of substrate hydrolysis in a dose-dependent manner compared to the untreated control.

By the 20 min endpoint, EC-ETAC significantly reduced DPP-IV activity, with the 100 µg/mL dose decreasing activity to 61.37 ± 2.42% of the control value (Figure 5B). Notably, dieckol displayed higher molar potency; at a concentration of 25 µM, dieckol suppressed in situ activity to 53.89 ± 2.42% (Figure 5E). This level of inhibition was statistically comparable to that of the clinical reference, Diprotin A, which reduced activity to 52.55 ± 2.54% at the same time point.

A comparison of the trends reveals that dieckol achieves a more rapid and robust suppression of DPP-IV at lower molar concentrations than the total extract. For instance, even at a low dose of 10 µM, dieckol maintained activity at 65.39 ± 1.89%, which is comparable to the effect of 50 µg/mL of EC-ETAC (66.83 ± 1.22%). These kinetic results demonstrate that dieckol effectively reaches and inhibits the DPP-IV enzyme situated on the apical membrane of intestinal cells, mirroring the high potency observed in the previous cell-free biochemical assays and suggesting strong potential for regulating incretin hormone degradation.

To determine whether this reduction in activity was a result of altered protein synthesis, the total protein expression of DPP-IV was quantified via Western blot (Figure 5C,F). Interestingly, neither EC-ETAC nor dieckol induced any significant change in the expression levels of the DPP-IV protein, with the band intensities remaining nearly identical to the untreated control across all concentrations. These results indicate that the antidiabetic potential of EC-ETAC and dieckol is mediated through the direct competitive or non-competitive inhibition of the enzyme’s catalytic activity rather than through the downregulation of its expression at the translational level.

### 2.7. EC-ETAC and Dieckol Ameliorate Hepatic Insulin Resistance Through AMPK Activation

**Figure 6:**
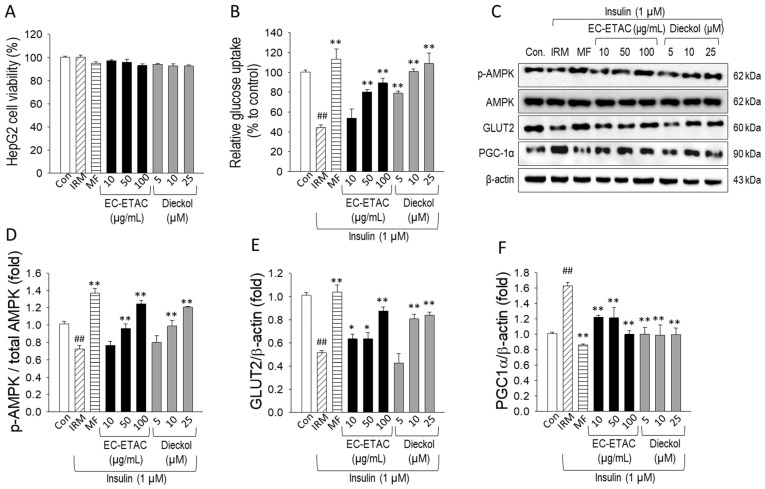
Effects of EC-ETAC and dieckol on glucose metabolism in Insulin-Resistant HepG2 cells. (**A**) Cell viability of HepG2 cells following treatment with EC-ETAC, dieckol, and 1 mM Metformin (MF) under IRM conditions. (**B**) Relative glucose uptake expressed as a percentage of the untreated control. (**C**) Representative Western blot bands and (**D**–**F**) relative quantification of p-AMPK/Total AMPK, GLUT2, and PGC-1α protein levels. Insulin resistance was induced using high-dose insulin (IRM), and Metformin (MF) served as the positive control. Results are expressed as mean ± SD (n = 3). Statistical significance is indicated by * (*p* < 0.05) and ** (*p* < 0.001) compared to the IRM group. Data are presented as mean ± SD (n = 3). Statistical significance compared to the control is indicated by * *p* < 0.05, ** *p* < 0.001 vs. IRM group; ## *p* < 0.001 vs. untreated control as determined by one-way ANOVA with Dunnett’s post hoc test.

To investigate the effects of EC on hepatic glucose metabolism, an Insulin Resistance Model (IRM) was established in HepG2 cells by treatment with 1 µM insulin for 24 h, resulting in a 60% reduction in glucose uptake capacity. Preliminary MTT assays confirmed that the induction of IRM, as well as subsequent treatments with EC-ETAC (10–100 µg/mL), dieckol (5–25 µM), and the positive control Metformin (1 mM), exerted no significant cytotoxicity, with cell viability remaining statistically comparable to the untreated control (Figure 6A).

The functional recovery of glucose metabolism was assessed via a glucose uptake assay (Figure 6B). Induction of the IRM significantly suppressed glucose uptake to 44.28 ± 2.67% of the control. However, treatment with EC-ETAC and dieckol restored uptake in a dose-dependent manner. High-dose EC-ETAC (100 µg/mL) increased uptake to 89.31 ± 4.96%, while dieckol (25 µM) effectively reversed the insulin resistance, restoring glucose uptake to 108.97 ± 10.74%, which was statistically comparable to the 1 mM Metformin group (113.52 ± 10.38%).

Western blot analysis (Figure 6C) was performed to elucidate the signaling pathways involved. In the IRM group, the phosphorylation (activation) of AMPK was significantly reduced to 0.72 ± 0.04-fold of the control, consistent with the impaired energy sensing typically seen in insulin-resistant states. Treatment with dieckol (25 µM) significantly stimulated AMPK phosphorylation, reaching 1.21 ± 0.01-fold, surpassing the levels of the untreated control (Figure 6D) and performing comparably to the 1 mM Metformin group (1.37 ± 0.06-fold). The expression of GLUT2 was also analyzed, as its membrane translocation and total expression are critical for maintaining hepatic glucose homeostasis; however, in insulin-resistant conditions, this vital transport mechanism is frequently compromised. GLUT2 was downregulated in the IRM group (0.51 ± 0.02) but was significantly restored by dieckol to 0.84 ± 0.03-fold (Figure 6E). Notably, this restoration by 25 µM dieckol was highly effective, approaching the levels observed in the Metformin-treated cells (1.04 ± 0.06-fold).

Furthermore, the expression of PGC-1α was examined, as it serves as a key transcriptional coactivator regulating energy metabolism and gluconeogenesis; its dysregulation is a hallmark of hepatic metabolic dysfunction. While PGC-1α levels were significantly elevated in the IRM group (1.63 ± 0.05-fold), likely as a compensatory response to insulin resistance and metabolic stress, treatment with dieckol and EC-ETAC effectively modulated these levels back toward the basal state. Notably, 25 µM dieckol restored PGC-1α expression to 0.96 ± 0.03-fold, showing a more precise normalization compared to the 1 mM Metformin group, which reduced expression to 0.86 ± 0.01-fold (Figure 6F). These results indicate that dieckol ameliorates hepatic insulin resistance and restores metabolic homeostasis primarily through the activation of the AMPK signaling pathway, subsequently enhancing GLUT2-mediated glucose uptake and regulating downstream metabolic coactivators.

## 3. Discussion

In this experiment, EC-ETAC and its major phlorotannin, dieckol, demonstrate multi-compartmental antidiabetic activity that simultaneously targets intestinal carbohydrate digestion, glucose absorption, incretin preservation, and hepatic insulin sensitivity. Absolute quantification by HPLC–DAD confirmed that dieckol is present in the EC-ETAC extract at 36.29 ± 1.65 mg/g (3.63% *w*/*w*). This concentration is within the range reported for commercial *E. cava* extracts (13.3–592 mg/g) and significantly higher than that found in raw algal tissue [31,32,33]. Chowdhury et al. (2011) developed a validated HPLC method and quantified dieckol in *E. cava* tissues, reporting a content of 1.82 mg/g-dry tissue in mature thalli, with dieckol and phlorofucofuroeckol-A together representing approximately 54% of the crude phlorotannins, further confirming dieckol as a key metabolite [31]. Goo et al. (2010) also quantitatively determined that dieckol was the most abundant phlorotannin in both ethanol and ethyl acetate extracts of Ecklonia species [34]. Dieckol has been reported to possess potent antidiabetic properties, including inhibition of α-glucosidase and α-amylase activities in vitro, as well as reduction in blood glucose levels, serum insulin, and body weight in type 2 diabetic db/db mouse models through activation of the AMPK and Akt signaling pathways [24,35,36,37]. However, previous studies have not investigated its effects on intestinal glucose transporter regulation, direct DPP-IV inhibition in cellular environments, or the structural basis of DPP-IV binding. The present study fills these gaps by demonstrating SGLT1/GLUT2 downregulation, in situ DPP-IV inhibition, and molecular docking evidence of dieckol binding to the DPP-IV catalytic site. Together with our findings on AMPK-mediated hepatic glucose uptake restoration, these coordinated multi-compartmental actions exceed the single-target profiles of acarbose, Diprotin A, and metformin, offering a mechanistic basis for superior postprandial glycemic control and hepatic metabolic restoration.

EC-ETAC inhibited α-glucosidase (IC_50_ = 2.2 ± 0.2 µg/mL) and α-amylase (IC_50_ = 41.0 ± 1.2 µg/mL) with potencies 26-fold and 6-fold greater than acarbose, respectively. Pure dieckol exhibited potent inhibitory activity against both enzymes (α-glucosidase IC_50_ = 2.213 ± 0.04 µM; α-amylase IC_50_ = 156.87 ± 0.124 µM), confirming it as a key contributor to the observed activity of the extract. Notably, the IC_50_ values obtained in this study were substantially lower (indicating greater potency) than those previously reported for dieckol (0.24 mM and 0.66 mM, respectively) [24], which may be attributed to differences in assay conditions, including enzyme source, substrate concentration, or incubation parameters. The hexameric structure of dieckol, with its high density of hydroxyl groups, enables extensive hydrogen bonding within the enzyme active sites, delaying oligosaccharide hydrolysis at the brush border and reducing luminal glucose availability [38,39]. This inhibition in the intestinal lumen aligns with the postprandial glucose-lowering observed in streptozotocin-induced diabetic mice, where dieckol administration decreased the area under the curve from 483 to 259 mmol·min/L [24]. From a pharmacological perspective, the dieckol concentration in EC-ETAC (36.29 ± 1.65 mg/g) is sufficient to drive a substantial portion of the extract’s anti-diabetic activity. Based on the IC_50_ values of pure dieckol against α-glucosidase (2.21 µM) and DPP-IV (12.12 µM), the calculated dieckol content in the extract can account for a significant fraction of the observed enzyme inhibition.

In differentiated Caco-2 monolayers, EC-ETAC and dieckol further exerted a dual blockade by downregulating SGLT1 and GLUT2 protein expression to approximately 0.5-fold of control levels while suppressing 2-NBDG uptake by 46–53% over 120 min. This coordinated reduction in transporter abundance directly lowers the maximal transport capacity (V_max) for apical sodium-dependent glucose uptake via SGLT1 and basolateral facilitated diffusion via GLUT2, thereby attenuating overall transepithelial glucose flux across the intestinal epithelium [40,41]. Dieckol and EC-ETAC may suppress glucose transport through multiple mechanisms, including transcriptional repression of SGLT1 and GLUT2 genes, interference with transcription factors such as HNF-1α, and modulation of AMPK-mediated signaling pathways that regulate transporter trafficking, membrane localization, and protein stability [42]. Additionally, the high hydroxyl content of phlorotannins may enable direct binding to the transporter proteins, promoting their internalization or accelerating degradation [43]. In contrast, acarbose failed to downregulate these transporters or reduce 2-NBDG uptake. Acarbose acts exclusively as a competitive inhibitor of α-glucosidase in the intestinal lumen, slowing the final step of carbohydrate digestion into absorbable monosaccharides without directly interacting with glucose transporters or altering their expression [44]. Because the 2-NBDG uptake assay measures the transport of a fluorescent glucose analog across the cell membrane independently of luminal digestion, acarbose has minimal impact on intracellular accumulation of 2-NBDG once glucose or its analog is already available. This demonstrates that dieckol’s actions extend far beyond simple enzymatic blockade, involving direct regulation of the glucose transport mechanism itself. Polyphenols commonly attenuate intestinal glucose transport through both direct interaction with transporters and transcriptional repression; for instance, anthocyanin-rich berry extracts reduce SGLT1 and GLUT2 mRNA and protein abundance in Caco-2 cells, while quercetin, apigenin, and chrysin decrease GLUT2 and GLUT5 expression by 75–90% [45,46,47,48,49,50]. Mulberry leaf polyphenols similarly suppress the SGLT1–GLUT2 pathway at the mRNA level, lowering glucose absorption [51,52]. The present findings extend these observations to marine phlorotannins, establishing that dieckol and EC-ETAC attenuate maximal glucose transport capacity through reduced SGLT1 and GLUT2 protein level mechanistic feature not exhibited by synthetic α-glucosidase inhibitors, which leave transporter expression unaltered.

EC-ETAC and dieckol also inhibited DPP-IV activity in cell-free assays (dieckol IC_50_ = 12.12 ± 0.021 µM) and in situ in Caco-2 monolayers, where 25 µM dieckol reduced activity to 53.89% of control, comparable to Diprotin A. Diprotin A was selected for this study as it is a well-characterized, specific competitive inhibitor of DPP-IV, making it a standard reference for validating catalytic blockade in both biochemical and cell-based models. While Diprotin A provides a robust mechanistic benchmark, future comparative studies with clinically approved gliptins, such as sitagliptin, would further enhance the translational relevance of these findings. Notably, despite the significant reduction in enzymatic activity, DPP-IV protein expression remained unchanged. This result demonstrates that the observed effects are mediated through direct catalytic-site blockade rather than transcriptional suppression. This functional inhibition without alteration of total protein concentration is characteristic of direct enzyme inhibitors that occupy the active site and prevent substrate access, a mechanism commonly observed with natural polyphenols that act competitively on DPP-IV [53,54]. In the present study, the high hydroxyl density of dieckol enables multi-point interactions within the catalytic pocket, physically obstructing substrate binding and cleavage without triggering any feedback regulation of DPP-IV gene expression or protein turnover. Molecular docking revealed exceptional binding affinity (−10.396 kcal/mol; predicted *Kd* ≈ 23.97 nM), with dieckol occupying the S1 and S2 subpockets, forming hydrogen bonds and salt bridges with Glu205, Glu206, Arg125, and Arg358, and occluding the catalytic triad (Ser630, His740) through π-stacking with Tyr547, Tyr662, and Tyr666. These interactions surpass the binding energies reported for sitagliptin (−8.1 kcal/mol) and other gliptins (−7.3 to −9.5 kcal/mol), explaining the potent preservation of GLP-1 without altering enzyme abundance [55,56,57,58]. Similar patterns have been reported for other dietary polyphenols such as curcumin, resveratrol, and selected flavonoids, which inhibit DPP-IV enzymatic activity in both cell-free and cellular systems while leaving protein levels unaltered, confirming that the observed effect is due to direct catalytic occlusion rather than any change in enzyme synthesis or degradation [53,59,60,61]. Although brown seaweed supplementation improves incretin activity and glycemic indices in meta-analyses [62], the present data provide the first direct demonstration of dieckol’s high affinity engagement with DPP-IV. These findings position dieckol as a natural DPP-IV inhibitor with a mechanism distinct from that of synthetic gliptins.

In insulin-resistant HepG2 cells, 25 µM dieckol restored glucose uptake to 108.97 ± 10.74% of control, comparable to 1 mM metformin. This rescue occurred through AMPK phosphorylation (1.21-fold), normalization of GLUT2 expression (0.84-fold), and restoration of PGC-1α to basal levels (0.96-fold). In insulin resistance, suppressed AMPK permits PGC-1α-driven gluconeogenesis and reduced GLUT2-mediated glucose influx; AMPK activation reverses this cascade by inhibiting gluconeogenic transcription and promoting GLUT2 membrane availability [63,64,65,66]. These findings mirror documented effects in db/db mice, where dieckol increased AMPK/Akt phosphorylation and improved systemic insulin sensitivity [67,68]. Furthermore, EC extracts have been reported to activate hepatic AMPK/SIRT1 signaling and thereby reduce lipogenic stress [69,70], which may be attributed to the diverse array of bioactive constituents present in the EC extract. For instance, previous reports have identified the presence of fucoxanthin in EC extracts, a carotenoid known to independently activate the AMPK pathway and enhance glucose uptake [71,72]. Although pure dieckol independently recapitulated the key effects observed with the crude extract, the possibility of additive or synergistic contributions from other constituents cannot be entirely excluded.

Collectively, dieckol integrates inhibition of carbohydrate-digesting enzymes, downregulation of intestinal glucose transporters, DPP-IV inhibition, and AMPK-mediated hepatic restoration into a single multi-targeted framework that addresses postprandial hyperglycemia and insulin resistance at multiple physiological checkpoints. This comprehensive mechanism offers clear advantages over current pharmacotherapies, which typically target only one or two pathways. The findings align with clinical evidence that brown seaweed supplementation (>1000 mg/day) lowers postprandial glucose, HbA1c, and HOMA-IR [62]. The absence of cytotoxicity at efficacious concentrations further supports the translational potential of dieckol as a functional food component or lead compound for type 2 diabetes management. Nevertheless, certain limitations should be acknowledged. The use of Caco-2 and HepG2 cell lines, while standard for mechanistic studies, cannot fully reproduce the complex inter-organ signaling and metabolic flux present in living organisms. The present investigation focused on selected protein markers, leaving broader systemic effects of dieckol—such as those on the gut microbiome, incretin-secreting cells, or peripheral tissues—unexplored. Furthermore, active GLP-1 levels were not directly measured, and the achievable plasma or intestinal concentrations of dieckol following oral consumption remain unknown. Therefore, future studies are needed to validate these mechanisms in vivo under conditions of chronic hyperglycemia and to assess oral bioavailability, thereby confirming systemic efficacy and long-term safety.

## 4. Materials and Methods

### 4.1. Materials and Reagents

EC powder was provided by the National Marine Biodiversity Institute of Korea (MABIK). Dieckol (CS-0527665, purity ≥ 99.81%) was purchased from ChemScene (Monmouth Junction, NJ, USA). Human colorectal adenocarcinoma (Caco-2) and hepatocellular carcinoma (HepG2) cell lines were obtained from the American Type Culture Collection (ATCC; Manassas, VA, USA). Dulbecco’s Modified Eagle’s Medium (DMEM) was sourced from HyClone (Waltham, MA, USA), while fetal bovine serum (FBS), trypsin-EDTA, and antibiotics were procured from Gibco BRL (Grand Island, NY, USA). For biochemical and cellular assays, α-glucosidase, α-amylase, p-nitrophenyl-β-D-glucopyranoside (pNPG), DPP-IV enzyme, acarbose, Diprotin A, streptozotocin, metformin, dinitrosalicylic acid (DNS), 2-NBDG, and 3-(4,5-dimethylthiazol-2-yl)-2,5-diphenyltetrazolium bromide (MTT) were purchased from Sigma-Aldrich Co. (St. Louis, MO, USA). Primary antibodies for Western blot analysis were acquired from Santa Cruz Biotechnology (Santa Cruz, CA, USA) and Abcam (Cambridge, UK).

### 4.2. Sequential Solvent Extraction and Fractionation of EC

**Figure 7:**
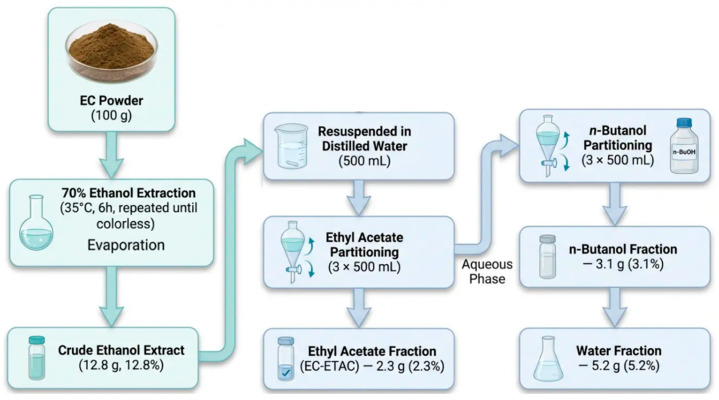
Schematic workflow of the sequential solvent extraction and fractionation process of *Ecklonia cava* based on solvent polarity.

The extraction and fractionation process were performed based on solvent polarity, as illustrated in Figure 7. Dried EC powder (100 g) was first extracted with 2 L of 70% ethanol at room temperature for 6 h in a shaker incubator (35 °C). The mixture was filtered through Whatman No. 41 filter paper, and the residue was re-extracted under identical conditions until the extract became colorless. The combined filtrates were concentrated under reduced pressure using a rotary evaporator (Büchi, Flawil, Switzerland) at 45 °C and subsequently freeze-dried to obtain the crude ethanol extract (yield: 12.8 g, 12.8% *w*/*w* relative to starting dry powder). The dried ethanol extract (12.8 g) was then resuspended in 500 mL of distilled water and sequentially partitioned with equal volumes of ethyl acetate (3 × 500 mL), followed by n-butanol (3 × 500 mL). Each partitioning step involved vigorous shaking for 10 min, after which the phases were allowed to separate. The organic phases (ethyl acetate and n-butanol) were collected sequentially, concentrated under reduced pressure, and freeze-dried to yield the ethyl acetate fraction (EC-ETAC; yield: 2.3 g, 2.3% *w*/*w*) and the n-butanol fraction (yield: 3.1 g, 3.1% *w*/*w*), respectively. The remaining aqueous phase was freeze-dried to obtain the water fraction (yield: 5.2 g, 5.2% *w*/*w*). All fractions were stored at −80 °C until further analysis and bioassay. The total recovery relative to the starting ethanol extract was 82.8% (10.6 g out of 12.8 g), with minor losses occurring during partitioning and transfer steps.

### 4.3. Enzymatic Inhibition Assay

The inhibitory potential of EC extracts and dieckol against glucose-regulating enzymes was evaluated using standardized spectrophotometric assays. For α-amylase inhibition, samples were pre-incubated with porcine pancreatic α-amylase (0.5 U/mL, pH 6.9) at 37 °C for 10 min, followed by a 15 min reaction with 1% starch. The reaction was terminated with DNS reagent at 90 °C for 5 min, and absorbance was recorded at 540 nm. For α-glucosidase inhibition, samples were mixed with yeast α-glucosidase (1 U/mL, pH 6.8), pre-incubated for 10 min at 37 °C, and reacted with 5 mM pNPG substrate for 15 min before measuring absorbance at 405 nm. Acarbose served as the reference standard for both carbohydrate-digesting assays. DPP-IV inhibitory activity was determined in 50 mM Tris-HCl buffer (pH 7.4). Samples were pre-incubated with DPP-IV enzyme (1 mU/µL) for 10 min at 37 °C, followed by the addition of 1 mM Gly-Pro-*p*-nitroanilide substrate. After a 30 min incubation at 37 °C, the release of *p*-nitroaniline was quantified at 405 nm using a microplate reader (Multiskan™ GO, Thermo Scientific™, Waltham, MA, USA). Diprotin A was employed as the positive control. All results were calculated as a percentage of inhibition relative to the non-treated control.

### 4.4. Phytochemical Identification by LC–MS/MS

To characterize the ethyl acetate fraction of EC (EC-ETAC), 60 mg of the extract was dissolved in 1 mL of methanol, centrifuged, and the resulting supernatant was filtered and diluted 10-fold prior to analysis. Chromatographic separation was performed using an ultra-high-resolution Q-TOF-LC–MS/MS system (Bruker maXis-HD, Bruker Daltonics, Billerica, MA, USA). Samples (1 µL) were injected onto a C18 column (2.1 × 100 mm, 1.7 µm) maintained at 40 °C. The mobile phase consisted of (A) 0.1% formic acid in water and (B) 0.1% formic acid in acetonitrile, delivered at 0.3 mL/min. The gradient elution progressed from 5% to 95% B over 25 min, followed by a 2 min hold at 95% B and re-equilibration to initial conditions. Full-scan mass spectrometry and tandem mass spectrometry (MS/MS) fragmentation were conducted in positive electrospray ionization (ESI) mode over an *m*/*z* range of 50–1500, with a mass resolution of 75,000 FWHM and mass accuracy < 600 ppb using internal calibration. Data were processed using Bruker Compass DataAnalysis 4.2 and TASQ 1.4 software. Compounds were tentatively identified by comparing accurate mass (<2 ppm error), isotopic pattern, and retention time against a custom phlorotannin library. Relative content was calculated using the peak area normalization method.

### 4.5. Absolute Quantification of Dieckol by HPLC–DAD

The dieckol content in EC-ETAC was determined using an HPLC–DAD system (Thermo Dionex Ultimate 3000, Thermo Fisher Scientific, Waltham, MA, USA) equipped with a Hypersil Gold C18 column (250 × 4.6 mm, 5 µm). The mobile phase consisted of (A) water with 0.05% phosphoric acid and (B) acetonitrile. A gradient elution was performed at 1.0 mL/min and 25 °C as follows: 0–3 min (0% B), 3–6 min (0–30% B), 6–18 min (30–45% B), 18–19 min (45–70% B), 19–23 min (70% B), 23–24 min (70–0% B), and 24–30 min (0% B). Detection was at 230 nm with an injection volume of 10 µL.

A dieckol stock solution (1000 mg/L) was prepared by dissolving 2 mg of pure dieckol in 200 µL DMSO and diluting with 50% methanol to 2 mL. This was further diluted to 50 mg/L, and five calibration standards (1, 5, 10, 25, and 50 mg/L) were prepared by serial dilution with 50% methanol. EC-ETAC sample (10 mg) was dissolved in 10 mL of 70% methanol, sonicated for 15 min, and filtered (0.45 µm). Each standard was injected once due to limited standard availability, while EC-ETAC samples were analyzed in duplicate (*n* = 2). The calibration curve was constructed by plotting peak area against concentration, and dieckol content was calculated using the regression equation. Results are expressed as mg dieckol per g of extract (mg/g).

### 4.6. Molecular Docking Analysis

Molecular docking was performed to elucidate the binding interaction between dieckol and the DPP-IV enzyme. The three-dimensional structure of dieckol was sketched using ChemDraw Professional 16.0 (PerkinElmer, Waltham, MA, USA) and converted into a 3D format, with energy minimization conducted via the YASARA program (YASARA Biosciences GmbH, Vienna, Austria). The crystal structure of human DPP-IV (PDB ID: 1WCY) was retrieved from the RCSB Protein Data Bank and processed using PyMOL 2.4.0 (Schrödinger, LLC, New York, NY, USA). Preparation involved the removal of water molecules and heteroatoms, the addition of polar hydrogen atoms, and the optimization of amino acid side chains. Docking simulations were executed using the YASARA AutoDock plugin (YASARA version 21.12.19.L.64, YASARA Biosciences, Vienna, Austria) to identify the most favorable binding conformations based on the lowest binding energy and inhibition constants. The resulting ligand-protein complexes were visualized and analyzed using Discovery Studio Visualizer (Dassault Systèmes, San Diego, CA, USA) and PyMOL to identify key intermolecular interactions, including hydrogen bonding, hydrophobic contacts, and π-stacking within the active site of the DPP-IV enzyme.

### 4.7. Cell Culture Condition

Caco-2 and HepG2 cells were maintained in DMEM supplemented with 10% FBS and 1% penicillin-streptomycin at 37 °C in a 5% CO_2_ humidified atmosphere. For experiments, cells were seeded at a density of 1 × 10^5^ cells/well in 12-well plates. HepG2 cells were utilized for metabolic assays 48 h post-seeding. Meanwhile, Caco-2 cells were subjected to a 21-day post-confluence differentiation period, with medium replacement every two days, to establish a mature intestinal monolayer characterized by brush-border enzyme expression. Prior to treatment, cells were transitioned to serum-free DMEM for 24 h. Subsequently, cells were incubated with EC-ETAC or dieckol for 48 h, washed with PBS, and harvested for Western blot and glucose uptake analyses.

### 4.8. Cell Cytotoxicity

The viability of Caco-2 and HepG2 cells following treatment was evaluated using the MTT assay. Cells were seeded in 96-well plates at a density of 1 × 10^4^ cells/well and incubated for 24 h to allow for cell attachment. Subsequently, the medium was replaced with various concentrations of EC-ETAC or dieckol, and the plates were incubated for an additional 24 or 48 h. Following treatment, 20 µL of MTT solution (5 mg/mL) was added to each well, and the cells were incubated at 37 °C for 4 h to facilitate the formation of formazan crystals. The supernatant was then carefully aspirated, and the resulting crystals were dissolved in 150 µL of DMSO. Absorbance was measured at 570 nm using a microplate reader. Cell viability was expressed as a percentage relative to the untreated control group to determine the non-toxic dose range for subsequent experiments.

### 4.9. Intestinal Glucose Transport Assay

To evaluate the impact of EC-ETAC and dieckol on intestinal glucose absorption, uptake assays were performed using the fluorescent analog 2-NBDG. Differentiated Caco-2 cells were seeded in 96-well black-walled plates and grown for 21 days post-confluence to ensure a mature brush-border phenotype. Prior to treatment, cells were subjected to glucose starvation in glucose-free DMEM for 2 h. The cells were then incubated with samples for 48 h, followed by the addition of 100 µM 2-NBDG for 30 min at 37 °C. Intracellular fluorescence was quantified using a microplate reader at excitation and emission wavelengths of 485 nm and 535 nm, respectively.

### 4.10. In Situ DPP-IV Activity in Caco-2 Monolayers

To evaluate the inhibitory effect of EC-ETAC and dieckol in a physiological cellular environment, in situ DPP-IV activity was measured using 21-day differentiated Caco-2 cells. Monolayers were washed with pre-warmed PBS (pH 7.4) and pre-incubated with varying concentrations of samples for 20 min at 37 °C. The enzymatic reaction was initiated by adding 1 mM Gly-Pro-p-nitroanilide substrate directly to the live cell wells. Kinetic monitoring and endpoint activity were recorded at an absorbance wavelength of 405 nm using a multimode microplate reader. Readings were taken at 5 min intervals (5, 10, and 15 min) to determine reaction velocity, followed by a final measurement at 20 min to calculate the percentage of inhibition. To account for variations in cell density, the final activity was normalized against the total protein content of each well, as determined by a BCA protein assay kit, and expressed relative to the non-treated control.

### 4.11. Hepatic Insulin Resistance Model (IRM) and Glucose Uptake

To investigate the therapeutic potential of dieckol in a diabetic-like state, an Insulin Resistance Model (IRM) was established in HepG2 cells. Insulin resistance was induced by treating cells with a high concentration of insulin (1 µM) for 24 h, achieving a 60% reduction in glucose uptake capacity compared to normal control cells. Following the induction period, the IRM cells were treated with EC-ETAC, dieckol, or Metformin (1 mM) as a positive control for an additional 24 h. To assess the recovery of insulin sensitivity, glucose uptake was stimulated with 100 nM insulin for 30 min, followed by the addition of 50 µM 2-NBDG. After a 30 min incubation, intracellular fluorescence was measured at 485/535 nm.

### 4.12. Western Blot Analysis

Following treatment, cells were washed with ice-cold PBS and lysed using RIPA buffer supplemented with protease and phosphatase inhibitors. Protein concentrations were determined using a BCA assay to ensure equal loading. Equal amounts of protein (20–30 µg) were separated by SDS-PAGE and transferred onto PVDF membranes. After blocking with 5% non-fat milk, membranes were incubated overnight at 4 °C with primary antibodies against SGLT1, GLUT2, DPP-IV, GLP-1, AMPK, and p-AMPK. Following incubation with HRP-conjugated secondary antibodies, protein bands were visualized via enhanced chemiluminescence. Images were captured using a Davinch-Chemi Imager™ (CAS400SM, Core Bio, Seoul, Republic of Korea). Densitometric analysis was conducted using ImageJ (ver. 1.54g) to quantify protein expression, with all target proteins normalized to β-actin. For AMPK activation, results were expressed as the ratio of phosphorylated to total protein.

### 4.13. Statistical Analysis

All experimental results are presented as the mean ± standard deviation (SD) from at least three independent experiments. Statistical analysis was performed using SigmaPlot^®^ 12.0 software (Systat Software Inc., San Jose, CA, USA). Significant differences between groups were assessed using one-way analysis of variance (ANOVA). For cell-based assays where treatments were compared to a single untreated control, Dunnett’s post hoc test was utilized. A *p*-value of less than 0.05 (*p* < 0.05) was considered to indicate a statistically significant difference.

## 5. Conclusions

EC-ETAC and its major phlorotannin dieckol exhibit potent multi-targeted antidiabetic effects. EC-ETAC showed superior inhibition of α-glucosidase (IC_50_ = 2.2 µg/mL) and α-amylase (IC_50_ = 41.0 µg/mL) compared with acarbose, while dieckol achieved IC_50_ values of 2.213 µM and 156.87 µM, respectively. In differentiated Caco-2 cells, both samples downregulated SGLT1 and GLUT2 protein expression and suppressed glucose uptake, effects not observed with acarbose. They also inhibited DPP-IV activity (dieckol IC_50_ = 12.12 µM) without altering protein abundance, supported by high-affinity binding (−10.396 kcal/mol) that occludes the catalytic triad (Ser630, His740). In insulin-resistant HepG2 cells, dieckol (25 µM) restored glucose uptake to 108.97% of control via AMPK activation (1.21-fold), normalization of GLUT2 (0.84-fold), and PGC-1α recalibration (0.96-fold), matching or exceeding 1 mM metformin. Collectively, dieckol provides comprehensive glycemic control through luminal enzyme inhibition, intestinal dual blockade, incretin preservation, and hepatic AMPK-mediated restoration, positioning *Ecklonia cava* phlorotannins as promising natural leads for type 2 diabetes management.

## Figures and Tables

**Table 1:** IC_50_ values for α-glucosidase, α-amylase and DPP-IV inhibitory effects of *E. cava* various extracts and acarbose and Diprotin A as positive controls in µg/mL.

No	Samples	α-Glucosidase	α-Amylase	DPP-IV
1	Ethanol extract	18.2 ± 1.2	581.0 ± 68.4	72.0 ± 11.3
2	Ethyl acetate extract	2.2 ± 0.2	41.0 ± 1.2	21.0 ± 1.4
3	n-Butanol extract	14.9 ± 1.6	281.0 ± 10.3	not detected
4	Water extract	121.4 ± 0.8	not detected	not detected
5	Acarbose	57.2 ± 13.0	250.6 ± 40.2	not tested
6	Diprotin A	not tested	not tested	6.7 ± 0.12

**Table 2:** Identification of major phlorotannins in *E. cava* ethyl acetate extract by LC–Q-TOF-MS/MS.

Peak No.	RT (min)	Compound Name	Formula	Observed *m*/*z*	Adduct	Error (ppm)
1	2.90	4′-[2,4-Dihydroxy-6-(2,4,6-trihydroxyphenoxy)phenoxy]-2,2′,4,6,6′-biphenylpentol	C_24_H_18_O_12_	499.0881	[M+H]^+^	2.0
2	3.52	Fucodiphlorethol G	C_24_H_18_O_12_	499.0878	[M+H]^+^	1.3
3	3.83	Fucophlorethol A	C_18_H_14_O_9_	375.0717	[M+H]^+^	1.8
4	8.36	Eckol	C_18_H_12_O_9_	373.0559	[M+H]^+^	1.3
5	8.62	7-Phloroeckol	C_24_H_16_O_13_	497.0721	[M+H]^+^	1.3
6	9.37	8,8′-Bieckol	C_36_H_22_O_18_	743.0879	[M+H]^+^	0.1
7	9.48	2-O-(2,4,6-trihydroxyphenyl)-6,6′-bieckol	C_42_H_26_O_21_	867.1042	[M+H]^+^	0.4
8	10.41	Dibenzodioxin-fucodiphloroethol	C_36_H_24_O_19_	745.1036	[M+H]^+^	0
9	12.34	Dieckol	C_36_H_22_O_18_	743.0877	[M+H]^+^	−0.3
10	14.62	Phlorofucofuroeckol A	C_30_H_18_O_15_	603.0768	[M+H]^+^	−0.2

## Data Availability

The original contributions presented in this study are included in the article and Supplementary Materials. Further inquiries can be directed to the corresponding author.

## Supplementary Materials

The following supporting information can be downloaded at: https://www.mdpi.com/article/10.3390/md24050174/s1. Supplementary Table S1 with complete MS/MS identification data for 16 compounds; Supplementary Table S2 with HPLC-DAD calibration curve and dieckol quantification; Supplementary Figures S1–S10 showing MS/MS spectra of the ten major phlorotannins listed in Table 2.

## References

[1] Huang Q., Li Y., Yu M., Lv Z., Lu F., Xu N., Zhang Q., Shen J., Zhu J., Jiang H. (2025). Global burden and risk factors of type 2 diabetes mellitus from 1990 to 2021, with forecasts to 2050. Front. Endocrinol..

[2] Standl E., Khunti K., Hansen T.B., Schnell O. (2019). The global epidemics of diabetes in the 21st century: Current situation and perspectives. Eur. J. Prev. Cardiol..

[3] Abdul Basith Khan M., Hashim M.J., King J.K., Govender R.D., Mustafa H., Al Kaabi J. (2020). Epidemiology of type 2 diabetes—Global burden of disease and forecasted trends. J. Epidemiol. Glob. Health.

[4] González P., Lozano P., Ros G., Solano F. (2023). Hyperglycemia and oxidative stress: An integral, updated and critical overview of their metabolic interconnections. Int. J. Mol. Sci..

[5] Uuh Narvaez J.J., Segura Campos M.R. (2022). Combination therapy of bioactive compounds with acarbose: A proposal to control hyperglycemia in type 2 diabetes. J. Food Biochem..

[6] Lillich F.F., Goorani S., Proschak E., Imig J.D. (2025). Multi-target Drugs to Treat Metabolic Diseases. Polypharmacology: Strategies for Multi-Target Drug Discovery.

[7] Hayes M.R., Borner T., De Jonghe B.C. (2021). The Role of GIP in the Regulation of GLP-1 Satiety and Nausea. Diabetes.

[8] Sharma A., Virmani T., Sharma A., Chhabra V., Kumar G., Pathak K., Alhalmi A. (2022). Potential effect of DPP-4 inhibitors towards hepatic diseases and associated glucose intolerance. Diabetes Metab. Syndr. Obes. Targets Ther..

[9] Singh A.-K., Yadav D., Sharma N., Jin J.-O. (2021). Dipeptidyl peptidase (DPP)-IV inhibitors with antioxidant potential isolated from natural sources: A novel approach for the management of diabetes. Pharmaceuticals.

[10] McKillop A.M., Stevenson C.L., Moran B.M., Abdel-Wahab Y.H., Flatt P.R. (2018). Tissue expression of DPP-IV in obesity-diabetes and modulatory effects on peptide regulation of insulin secretion. Peptides.

[11] Lee E.Y., Kim Y.W., Oh H., Choi C.S., Ahn J.H., Lee B.-W., Kang E.S., Cha B.S., Lee H.C. (2014). Anti-obesity effects of KR-66195, a synthetic DPP-IV inhibitor, in diet-induced obese mice and obese-diabetic ob/ob mice. Metabolism.

[12] Singh A.-K., Jatwa R., Purohit A., Ram H. (2017). Synthetic and phytocompounds based dipeptidyl peptidase-IV (DPP-IV) inhibitors for therapeutics of diabetes. J. Asian Nat. Prod. Res..

[13] Sakai Y., Chen G., Ni Y., Zhuge F., Xu L., Nagata N., Kaneko S., Ota T., Nagashimada M. (2020). DPP-4 Inhibition with Anagliptin Reduces Lipotoxicity-Induced Insulin Resistance and Steatohepatitis in Male Mice. Endocrinology.

[14] Hayes M., García-García M., Fitzgerald C., Lafarga T. (2015). Seaweed and milk derived bioactive peptides and small molecules in functional foods and cosmeceuticals. Biotechnology of Bioactive Compounds: Sources and Applications.

[15] Chellappan D.K., Chellian J., Rahmah N.S.N., Gan W.J., Banerjee P., Sanyal S., Banerjee P., Ghosh N., Guith T., Das A. (2023). Hypoglycaemic molecules for the management of diabetes mellitus from marine sources. Diabetes Metab. Syndr. Obes..

[16] Unnikrishnan P.S., Jayasri M.A. (2018). Marine algae as a prospective source for antidiabetic compounds—A brief review. Curr. Diabetes Rev..

[17] Reddy K.T.K., Rakesh K., Prathyusha S., Gupta J.K., Nagasree K., Lokeshvar R., Elumalai S., Prasad P.D., Kolli D. (2025). Revolutionizing diabetes care: The role of marine bioactive compounds and microorganisms. Cell Biochem. Biophys..

[18] Brizzi A., Rispoli R.M., Autore G., Marzocco S. (2025). Anti-inflammatory effects of algae-derived biomolecules in gut health: A review. Int. J. Mol. Sci..

[19] Manandhar B., Paudel P., Seong S.H., Jung H.A., Choi J.S. (2019). Characterizing eckol as a therapeutic aid: A systematic review. Mar. Drugs.

[20] Han E.J., Kim H.-S., Sanjeewa K., Herath K., Jeon Y.-J., Jee Y., Lee J., Kim T., Shim S.-Y., Ahn G. (2020). Eckol from *Ecklonia cava* suppresses immunoglobulin E-mediated mast cell activation and passive cutaneous anaphylaxis in mice. Nutrients.

[21] Park S.R., Kim J.H., Jang H.D., Yang S.Y., Kim Y.H. (2018). Inhibitory activity of minor phlorotannins from *Ecklonia cava* on α-glucosidase. Food Chem..

[22] Lee H.-A., Lee J.-H., Han J.-S. (2017). A phlorotannin constituent of *Ecklonia cava* alleviates postprandial hyperglycemia in diabetic mice. Pharm. Biol..

[23] Ryu J.-W., Lee M.S., Yim M.-J., Lee J.M., Lee D.-S., Kim Y.-M., Eom S.-H. (2023). α-Amylase and α-glucosidase inhibition effects of Korean edible brown, green, and red seaweed extracts. Fish. Aquat. Sci..

[24] Lee S.-H., Park M.-H., Heo S.-J., Kang S.-M., Ko S.-C., Han J.-S., Jeon Y.-J. (2010). Dieckol isolated from *Ecklonia cava* inhibits α-glucosidase and α-amylase in vitro and alleviates postprandial hyperglycemia in streptozotocin-induced diabetic mice. Food Chem. Toxicol..

[25] Almutairi M.G., Aldubayan K., Molla H. (2023). Effect of seaweed (*Ecklonia cava* extract) on blood glucose and insulin level on prediabetic patients: A double-blind randomized controlled trial. Food Sci. Nutr..

[26] Casas-Agustench P., Mínguez S., Brookes Z., Bescos R. (2025). Edible Algae Reduce Blood Pressure in Humans: A Systematic Review and Meta-Analysis of Randomised Controlled Trials. J. Hum. Nutr. Diet..

[27] Vaughan K., Ranawana V., Cooper D., Aceves-Martins M. (2022). Effect of brown seaweed on plasma glucose in healthy, at-risk, and type 2 diabetic individuals: Systematic review and meta-analysis. Nutr. Rev..

[28] McLaughlin C.M., Harnedy-Rothwell P.A., Lafferty R., Sharkey S., Parthsarathy V., Allsopp P.J., McSorley E.M., FitzGerald R.J., O’Harte F.P. (2021). Macroalgal protein hydrolysates from Palmaria palmata influence the ‘incretin effect’ in vitro via DPP-4 inhibition and upregulation of insulin, GLP-1 and GIP secretion. Eur. J. Nutr..

[29] Isara R., Gunathilaka M. (2025). Unveiling the Therapeutic Potential of Marine Algae-Derived Compounds. Seaweeds as Silent Healers: Insight into the Pharmacological Potential of Marine Algal Metabolites.

[30] Harnedy P.A., O’Keeffe M.B., FitzGerald R.J. (2015). Purification and identification of dipeptidyl peptidase (DPP) IV inhibitory peptides from the macroalga Palmaria palmata. Food Chem..

[31] Chowdhury M.T.H., Bangoura I., Kang J.-Y., Park N.G., Ahn D.-H., Hong Y.-K. (2011). Distribution of phlorotannins in the brown alga *Ecklonia cava* and comparison of pretreatments for extraction. Fish. Aquat. Sci..

[32] Lim S.-B., Lee J., Yang Y.-H., Son H., Yoo H.Y., Han J.-A. (2024). Development of a novel functional jelly with dieckol-rich extract from Eisenia bicyclis: Physicochemical, antioxidant, and sensory characterization. Food Chem. X.

[33] Kim D.-Y., Park H.-J., Yun C.-I., Kim Y.-J. (2024). Method development and validation of phloroglucinol and dieckol in *Ecklonia cava* using HPLC–DAD. Food Sci. Biotechnol..

[34] Goo H.R., Choi J.S., Na D.H. (2010). Quantitative determination of major phlorotannins in Ecklonia stolonifera. Arch. Pharmacal Res..

[35] Lee S.-H., Park M.-H., Kang S.-M., Ko S.-C., Kang M.-C., Cho S., Park P.-J., Jeon B.-T., Kim S.-K., Han J.-S. (2012). Dieckol isolated from *Ecklonia cava* protects against high-glucose induced damage to rat insulinoma cells by reducing oxidative stress and apoptosis. Biosci. Biotechnol. Biochem..

[36] Kang M.-C., Wijesinghe W., Lee S.-H., Kang S.-M., Ko S.-C., Yang X., Kang N., Jeon B.-T., Kim J., Lee D.-H. (2013). Dieckol isolated from brown seaweed *Ecklonia cava* attenuates type II diabetes in db/db mouse model. Food Chem. Toxicol..

[37] Yeo A.-R., Lee J., Tae I.H., Park S.-R., Cho Y.H., Lee B.H., Shin H.C., Kim S.H., Yoo Y.C. (2012). Anti-hyperlipidemic effect of polyphenol extract (Seapolynol™) and dieckol isolated from *Ecklonia cava* in in vivo and in vitro models. Prev. Nutr. Food Sci..

[38] Ogunwa T., Adeyelu T., Fasimoye R., Ayenitaju F. (2018). In silico analysis of interaction between seaweed-derived bioactive compounds and selected diabetes-related targets. Biomed. Chem. Res. Methods.

[39] Muthuraman A., Shaikh S.A., Ramesh M., Sikarwar M.S., Atta Ur R. (2021). Chapter 6—The structure–activity relationship of marine products for neuroinflammatory disorders. Studies in Natural Products Chemistry.

[40] Solini A., Rossi C., Mazzanti C.M., Proietti A., Koepsell H., Ferrannini E. (2017). Sodium-glucose co-transporter (SGLT) 2 and SGLT1 renal expression in patients with type 2 diabetes. Diabetes Obes. Metab..

[41] Yoshikawa T., Inoue R., Matsumoto M., Yajima T., Ushida K., Iwanaga T. (2011). Comparative expression of hexose transporters (SGLT1, GLUT1, GLUT2 and GLUT5) throughout the mouse gastrointestinal tract. Histochem. Cell Biol..

[42] Miyachi Y., Miyazawa T., Ogawa Y. (2022). HNF1A mutations and beta cell dysfunction in diabetes. Int. J. Mol. Sci..

[43] Ford L., Theodoridou K., Sheldrake G.N., Walsh P.J. (2019). A critical review of analytical methods used for the chemical characterisation and quantification of phlorotannin compounds in brown seaweeds. Phytochem. Anal..

[44] Rosak C., Mertes G. (2012). Critical evaluation of the role of acarbose in the treatment of diabetes: Patient considerations. Diabetes Metab. Syndr. Obes..

[45] Solverson P. (2020). Anthocyanin bioactivity in obesity and diabetes: The essential role of glucose transporters in the gut and periphery. Cells.

[46] Li Z., Tian J., Cheng Z., Teng W., Zhang W., Bao Y., Wang Y., Song B., Chen Y., Li B. (2023). Hypoglycemic bioactivity of anthocyanins: A review on proposed targets and potential signaling pathways. Crit. Rev. Food Sci. Nutr..

[47] Torres-Villarreal D., Camacho A., Milagro F.I., Ortiz-Lopez R., de la Garza A.L. (2017). Quercetin-3-O-glucoside improves glucose tolerance in rats and decreases intestinal sugar uptake in caco-2 cells. Nat. Prod. Commun..

[48] Abioye R.O., Okagu I.U., Udenigwe C.C. (2022). Targeting glucose transport proteins for diabetes management: Regulatory roles of food-derived compounds. J. Agric. Food Chem..

[49] Zakłos-Szyda M., Pietrzyk N., Kowalska-Baron A., Nowak A., Chałaśkiewicz K., Ratajewski M., Budryn G., Koziołkiewicz M. (2021). Phenolics-rich extracts of dietary plants as regulators of fructose uptake in Caco-2 cells via GLUT5 involvement. Molecules.

[50] Andrade N., Marques C., Andrade S., Silva C., Rodrigues I., Guardão L., Guimarães J.T., Keating E., Calhau C., Martel F. (2019). Effect of chrysin on changes in intestinal environment and microbiome induced by fructose-feeding in rats. Food Funct..

[51] Zhao Q., Yang J., Li J., Zhang L., Yan X., Yue T., Yuan Y. (2024). Hypoglycemic effect and intestinal transport of phenolics-rich extract from digested mulberry leaves in Caco-2/insulin-resistant HepG2 co-culture model. Food Res. Int..

[52] Li Q., Wang C., Liu F., Hu T., Shen W., Li E., Liao S., Zou Y. (2020). Mulberry leaf polyphenols attenuated postprandial glucose absorption via inhibition of disaccharidases activity and glucose transport in Caco-2 cells. Food Funct..

[53] Jia Y., Cai S., Muhoza B., Qi B., Li Y. (2023). Advance in dietary polyphenols as dipeptidyl peptidase-IV inhibitors to alleviate type 2 diabetes mellitus: Aspects from structure-activity relationship and characterization methods. Crit. Rev. Food Sci. Nutr..

[54] Fan J., Johnson M.H., Lila M.A., Yousef G., De Mejia E.G. (2013). Berry and citrus phenolic compounds inhibit dipeptidyl peptidase IV: Implications in diabetes management. Evid.-Based Complement. Altern. Med..

[55] Mathur V., Alam O., Siddiqui N., Jha M., Manaithiya A., Bawa S., Sharma N., Alshehri S., Alam P., Shakeel F. (2023). Insight into structure activity relationship of DPP-4 inhibitors for development of antidiabetic agents. Molecules.

[56] Ganguly G., Hussain A., Nayak A. In Silico Evaluation of DPP-4 Inhibitors: A Comparative Docking Approach. 2025, 5284350. https://ssrn.com/abstract=5284350.

[57] Abdella F.I., Alardan D., Daoud I., Alshammari N.S., Abdulrahman Alrashdi A., Boudriga S. (2026). Spiropyrrolizine derivatives as multitarget antidiabetic agents in obese type 2 diabetes: Integrated in silico, in vitro, and in vivo evaluation of DPP-4 inhibition and hepatic enzyme modulation. Future Med. Chem..

[58] Claus M.P., Ariantika L., Santoso F., Aryati W.D., Nepali K., Eden W.T. (2026). In Silico Studies of Sembukan (*Paederia scandens*) Secondary Metabolites as Anti Diabetes Against Multiple Target Protein. Trends Sci..

[59] Roney M., Dubey A., Issahaku A.R., Uddin M.N., Tufail A., Wilhelm A., Zamri N.B., Aluwi M.F.F.M. (2025). Insights from in silico exploration of major curcumin analogs targeting human dipeptidyl peptidase IV. J. Biomol. Struct. Dyn..

[60] Rohani, Febrina E., Wahyuni I.S., Levita J. (2023). Pharmacological and clinical studies of medicinal plants that inhibit dipeptidyl peptidase-IV. Drug Des. Devel. Ther..

[61] Liu Y.-H., Lin Y.-S., Sie Y.-Y., Wang C.-C., Chang C.-I., Hou W.-C. (2023). Vitisin B, a resveratrol tetramer from Vitis thunbergii var. taiwaniana, ameliorates impaired glucose regulations in nicotinamide/streptozotocin-induced type 2 diabetic mice. J. Tradit. Complement. Med..

[62] Kim Y.R., Park M.J., Park S.-Y., Kim J.Y. (2023). Brown seaweed consumption as a promising strategy for blood glucose management: A comprehensive meta-analysis. Nutrients.

[63] Agius L., Ford B.E., Chachra S.S. (2020). The metformin mechanism on gluconeogenesis and AMPK activation: The metabolite perspective. Int. J. Mol. Sci..

[64] Kim T., Davis J., Zhang A.J., He X., Mathews S.T. (2009). Curcumin activates AMPK and suppresses gluconeogenic gene expression in hepatoma cells. Biochem. Biophys. Res. Commun..

[65] Yu X., Meng Z., Fang T., Liu X., Cheng Y., Xu L., Liu X., Li X., Xue M., Li T. (2022). Empagliflozin inhibits hepatic gluconeogenesis and increases glycogen synthesis by AMPK/CREB/GSK3β signalling pathway. Front. Physiol..

[66] Kang O.-H., Shon M.-Y., Kong R., Seo Y.-S., Zhou T., Kim D.-Y., Kim Y.-S., Kwon D.-Y. (2017). Anti-diabetic effect of black ginseng extract by augmentation of AMPK protein activity and upregulation of GLUT2 and GLUT4 expression in db/db mice. BMC Complement. Altern. Med..

[67] Bai X., Pei R., Lei W., Zhao M., Zhang J., Tian L., Shang J. (2020). Antidiabetic effect of artemether in Db/Db mice involves regulation of AMPK and PI3K/Akt pathways. Front. Endocrinol..

[68] Bae U.-J., Jung E.-S., Jung S.-J., Chae S.-W., Park B.-H. (2018). Mulberry leaf extract displays antidiabetic activity in db/db mice via Akt and AMP-activated protein kinase phosphorylation. Food Nutr. Res..

[69] Eo H., Park J.E., Jeon Y.-j., Lim Y. (2017). Ameliorative effect of *Ecklonia cava* polyphenol extract on renal inflammation associated with aberrant energy metabolism and oxidative stress in high fat diet-induced obese mice. J. Agric. Food Chem..

[70] Wu Y., Jin X., Zhang Y., Liu J., Wu M., Tong H. (2023). Bioactive compounds from brown algae alleviate nonalcoholic fatty liver disease: An extensive review. J. Agric. Food Chem..

[71] Zhang Y., Xu W., Huang X., Zhao Y., Ren Q., Hong Z., Huang M., Xing X. (2018). Fucoxanthin ameliorates hyperglycemia, hyperlipidemia and insulin resistance in diabetic mice partially through IRS-1/PI3K/Akt and AMPK pathways. J. Funct. Foods.

[72] Kawee-Ai A., Kim A.T., Kim S.M. (2019). Inhibitory activities of microalgal fucoxanthin against α-amylase, α-glucosidase, and glucose oxidase in 3T3-L1 cells linked to type 2 diabetes. J. Oceanol. Limnol..

