# Supplementary material for: Antidiabetic Effects of Ecklonia cava and Dieckol via DPP-IV Inhibition and Glucose Transport Regulation
Source: Mar Drugs. 2026 May 12;24(5):174. doi: 10.3390/md24050174 (PMC13209096; doi:10.3390/md24050174)
Supplement: Supplementary file 1 [file marinedrugs-24-00174-s001.zip › marinedrugs-4267907-supplementary.pdf]

Supplementary Table of Contents:

**Supplementary Table S1**	Compounds identified in *Ecklonia cava* ethyl acetate extract by UPLC-Q-TOF-MS/MS in positive ion mode (sorted by retention time)	1
**Supplementary Table S2**	Calibration curve data and absolute quantification of dieckol in EC-ETAC extract by HPLC-DAD. A. calibration standard; B.EC-ETAC Sample Analysis; C. **HPLC-DAD Conditions**	2
**Supplementary Figure S1**	MS/MS spectrum of compound 1 (4'-[2,4-dihydroxy-6-(2,4,6-trihydroxyphenoxy)phenoxy]-2,2',4,6,6'-biphenylpentol) at RT 2.90 min	3
**Supplementary Figure S2**	MS/MS spectrum of compound 2 (fucodiphlorethol G) at RT 3.52 min	4
**Supplementary Figure S3**	MS/MS spectrum of compound 3 (fucophlorethol A) at RT 3.83 min	5
**Supplementary Figure S4**	MS/MS spectrum of compound 4 (eckol) at RT 8.36 min	6
**Supplementary Figure S5**	MS/MS spectrum of compound 5 (7-phloroeckol) at RT 8.62 min	7
**Supplementary Figure S6**	MS/MS spectrum of compound 6 (8,8'-bieckol) at RT 9.37 min	8
**Supplementary Figure S7**	MS/MS spectrum of compound 7 (2-O-(2,4,6-trihydroxyphenyl)-6,6'-bieckol) at RT 9.48 min	9
**Supplementary Figure S8**	MS/MS spectrum of compound 8 (dibenzodioxin-fucodiphloroethol) at RT 10.41 min	10
**Supplementary Figure S9**	MS/MS spectrum of compound 9 (dieckol) at RT 12.34 min	11
**Supplementary Figure S10**	MS/MS spectrum of compound 10 (phlorofucofuroeckol A) at RT 14.62 min	12

Supplementary Table S1. Compounds identified in Ecklonia cava ethyl acetate extract by UPLC-Q-TOF-MS/MS in positive ion mode (sorted by retention time).

No.	RT (min)	Compound Name	Molecular Formula	Observed m/z	Adduct	Mass Error (ppm)
1	2.90	4'-[2,4-Dihydroxy-6-(2,4,6-trihydroxyphenoxy)phenoxy]-2,2',4,6,6'-biphenylpentol	$C_{24}H_{18}O_{12}$	499.0881	$[M+H]^+$	2.0
2	3.52	Fucodiphlorethol G	$C_{24}H_{18}O_{12}$	499.0878	$[M+H]^+$	1.3
3	3.83	Fucophlorethol A	$C_{18}H_{14}O_9$	375.0717	$[M+H]^+$	1.8
4	8.36	Eckol	$C_{18}H_{12}O_9$	373.0559	$[M+H]^+$	1.3
5	8.62	7-Phloroeckol	$C_{24}H_{16}O_{13}$	497.0721	$[M+H]^+$	1.3
6	8.74	Eckstolonol	$C_{18}H_{10}O_9$	371.0404	$[M+H]^+$	1.7
7	8.94	Eckmaxol	$C_{36}H_{24}O_{19}$	745.1031	$[M+H]^+$	-0.6
8	9.13	2-O-(2,4,6-Trihydroxyphenyl)-6,6'-bieckol (isomer)	$C_{42}H_{26}O_{21}$	867.1039	$[M+H]^+$	-0.1
9	9.37	8,8'-Bieckol	$C_{36}H_{22}O_{18}$	743.0879	$[M+H]^+$	0.1
10	9.48	2-O-(2,4,6-Trihydroxyphenyl)-6,6'-bieckol	$C_{42}H_{26}O_{21}$	867.1042	$[M+H]^+$	0.4
11	10.41	Dibenzodioxin-fucodiphloroethol	$C_{36}H_{24}O_{19}$	745.1036	$[M+H]^+$	0.0
12	11.19	6,6'-Bieckol	$C_{36}H_{22}O_{18}$	743.0880	$[M+H]^+$	0.2
13	12.34	Dieckol	$C_{36}H_{22}O_{18}$	743.0877	$[M+H]^+$	-0.3
14	13.33	2',6,6'-Tris(3,5-dihydroxyphenoxy)-7-(2,4,6-trihydroxyphenoxy)-1,1'-bioxanthrene-2,4,4',7',9,9'-hexol	$C_{48}H_{31}O_{24}$	975.1246	$[M+H]^+$	-0.5
15	14.62	Phlorofucofuroeckol A	$C_{30}H_{18}O_{15}$	603.0768	$[M+H]^+$	-0.2
16	14.75	Unknown 974-B	$C_{48}H_{31}O_{24}$	975.1250	$[M+H]^+$	-0.1

Supplementary Table S2. Calibration curve data and absolute quantification of dieckol in EC-ETAC extract by HPLC-DAD

A. Calibration Standards

Standard	Concentration (mg/L)	Peak Area (mAU·min)	Retention Time (min)
Std 1	1	0.84	9.76
Std 2	5	3.33	9.76
Std 3	10	5.94	9.75
Std 4	25	10.32	9.76
Std 5	50	21.64	9.76

Regression equation: $y = 0.408x + 0.982$

Coefficient of determination (R^2): 0.993

Retention time (mean ± SD): 9.76 ± 0.01 min

B. EC-ETAC Sample Analysis

Replicate	Peak Area (mAU·min)	Calculated Conc. (mg/L)	Content (mg/g extract)
1	16.26	37.45	37.45
2	15.31	35.12	35.12

Mean ± SD (mg/g extract): 36.29 ± 1.65

% (w/w): 3.63%

C. HPLC-DAD Conditions

Column	Hypersil Gold C18 (250 × 4.6 mm, 5 μm)
Temperature	25°C
Mobile phase A	Water + 0.05% H_3PO_4
Mobile phase B	Acetonitrile
Flow rate	1.0 mL/min
Detection wavelength	230 nm
Injection volume	10 μL
Run time	30 min

Supplementary Figure S1–S10. Positive ion mode MS/MS spectra of the ten major phlorotannins identified in the EC-ETAC fraction by UPLC–Q-TOF–MS/MS.

4'-[2,4-Dihydroxy-6-(2,4,6-trihydroxyphenoxy)phenoxy]-2,2',4,6,6'-biphenylpentol

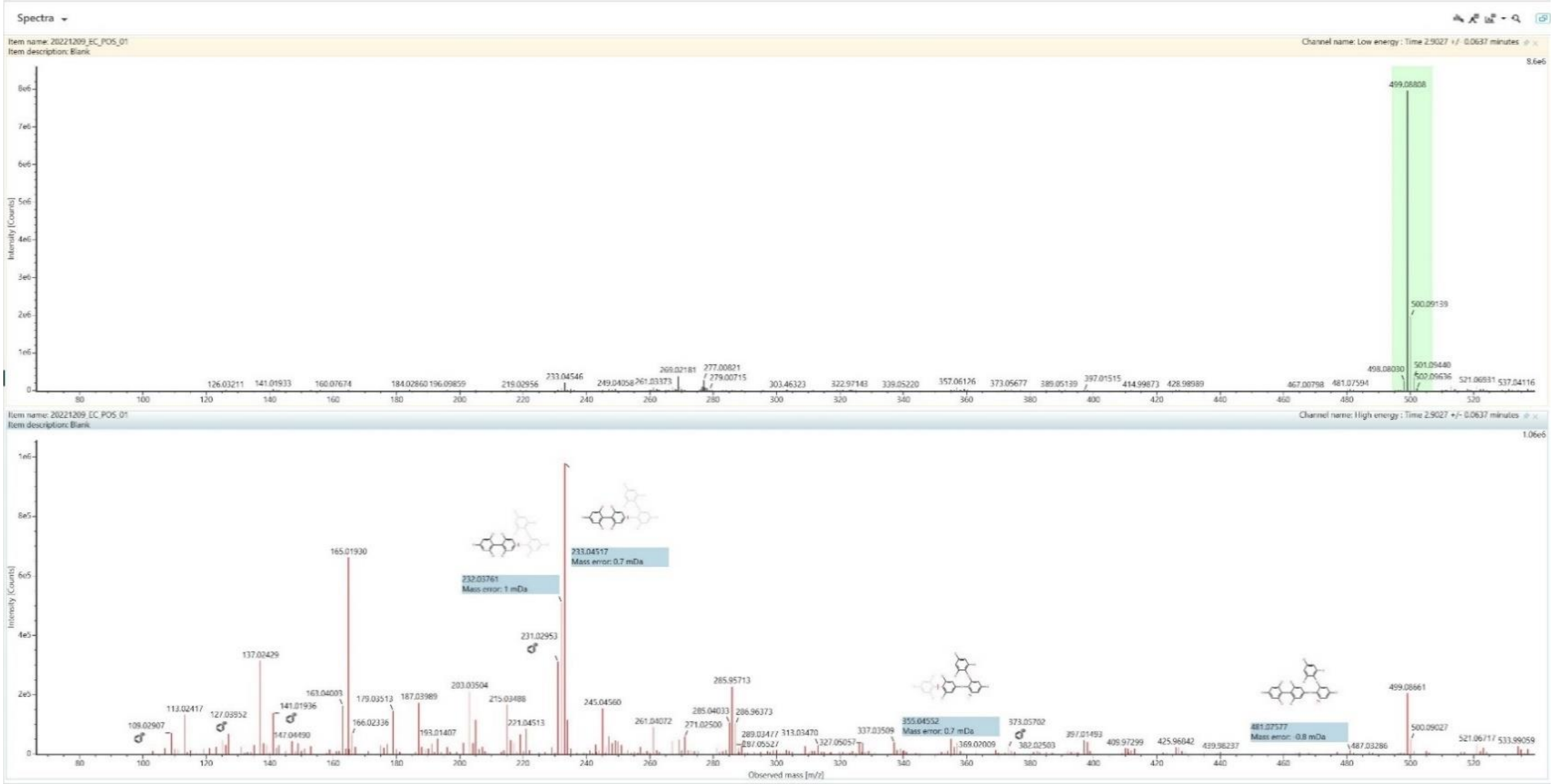

Figure S1. MS/MS spectrum of compound 1 (4'-[2,4-dihydroxy-6-(2,4,6-trihydroxyphenoxy)phenoxy]-2,2',4,6,6'-biphenylpentol) at RT 2.90 min. Precursor ion [M+H]$^{+}$ at *m/z* 499.0881.

Fucodiphlorethol G

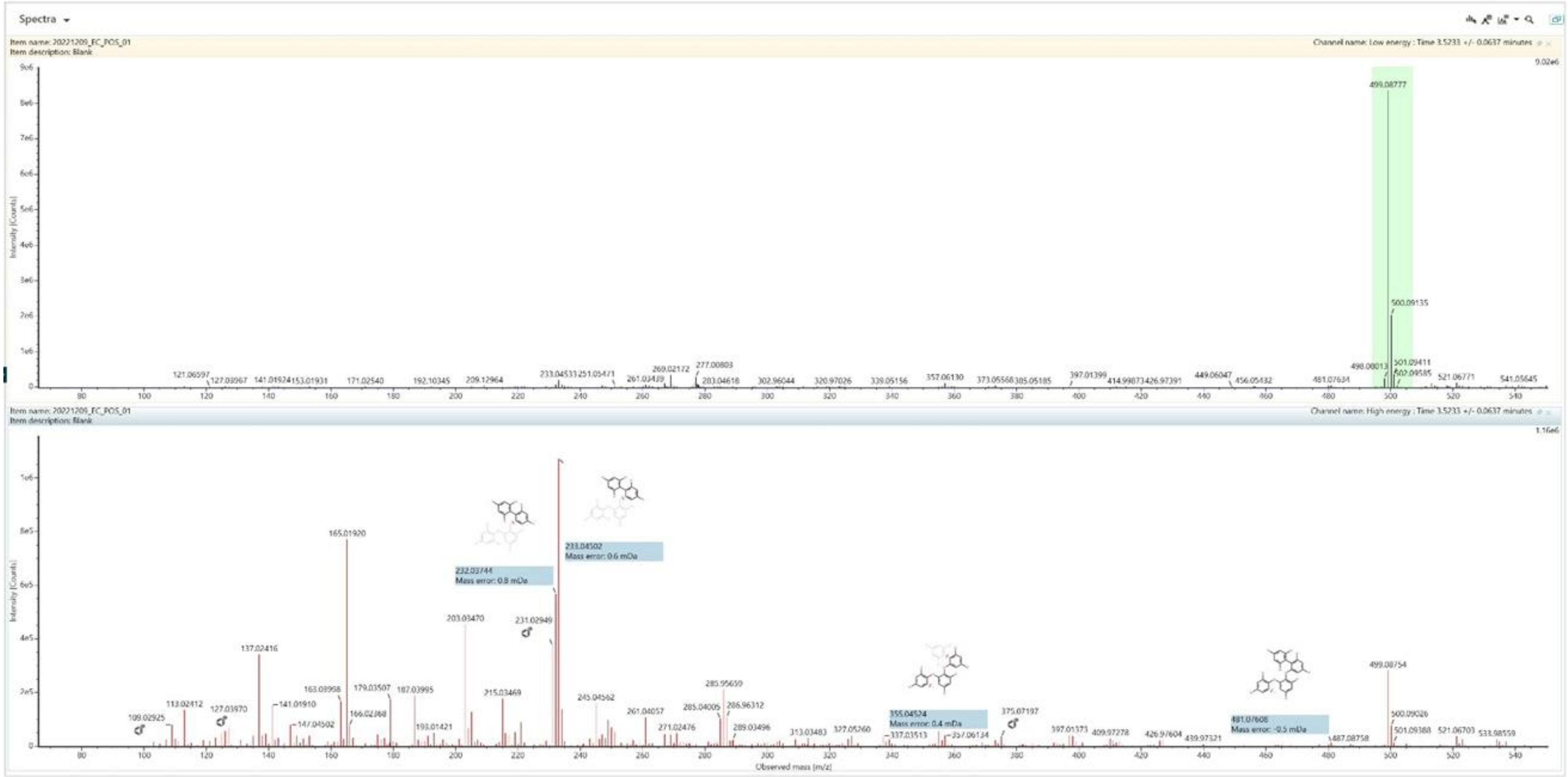

Figure S2. MS/MS spectrum of compound 2 (fucodiphlorethol G) at RT 3.52 min. Precursor ion $[M+H]^+$ at *m/z* 499.0878.

Fucophlorethol A

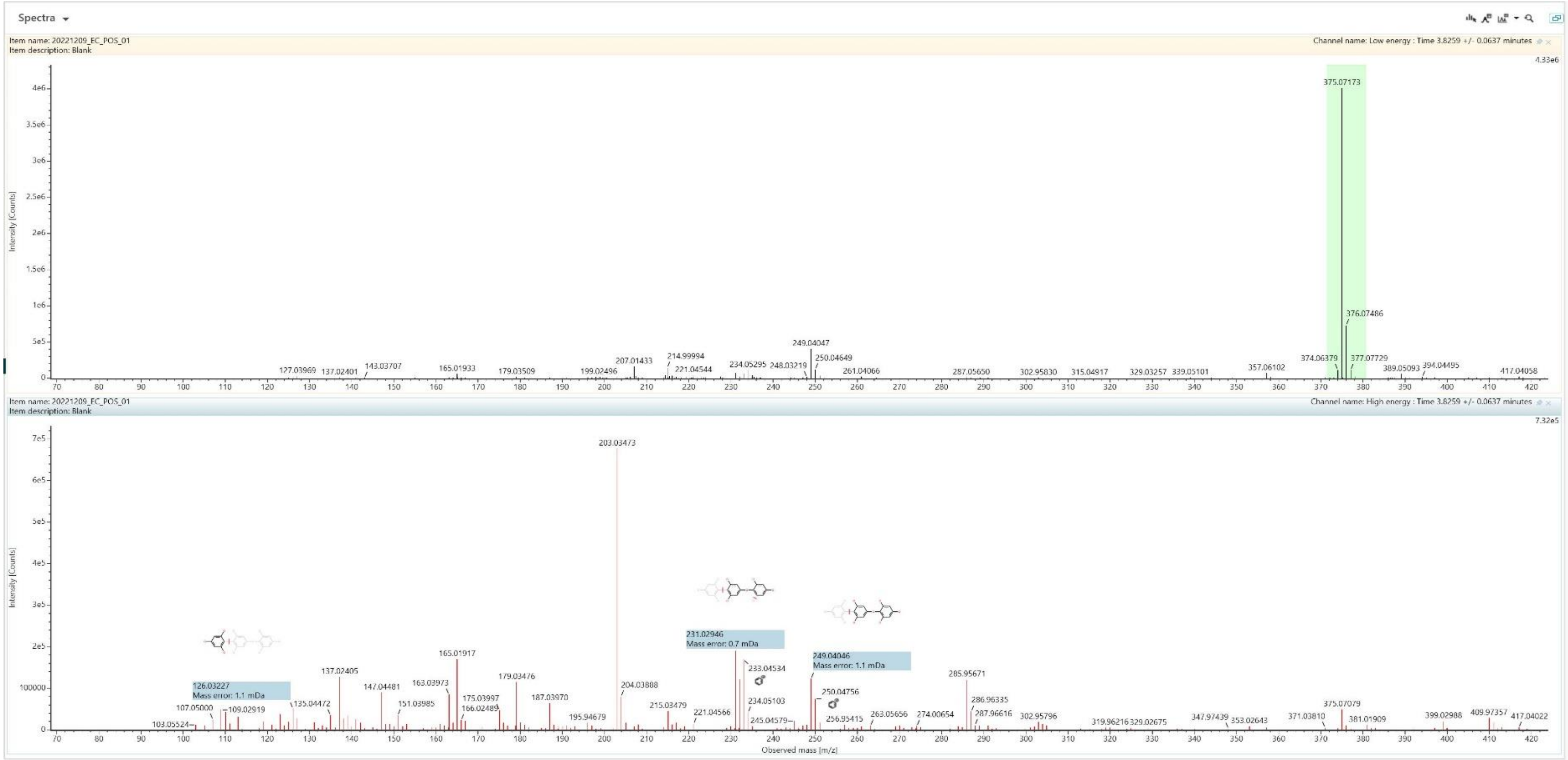

Figure S3. MS/MS spectrum of compound 3 (fucophlorethol A) at RT 3.83 min. Precursor ion $[M+H]^+$ at *m/z* 375.0717.

Eckol

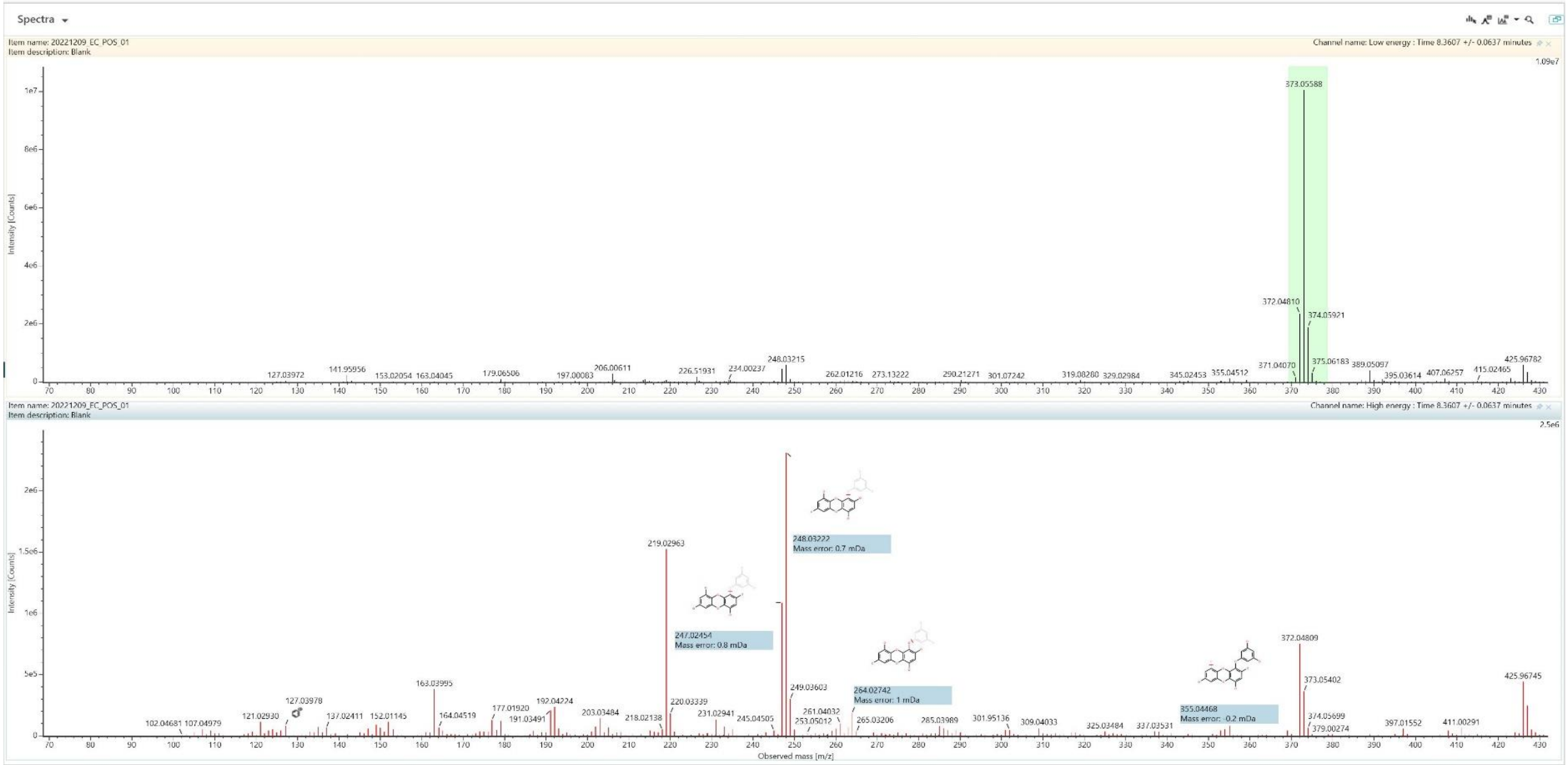

Figure S4. MS/MS spectrum of compound 4 (eckol) at RT 8.36 min. Precursor ion $[M+H]^+$ at *m/z* 373.0559.

7-Phloroeckol

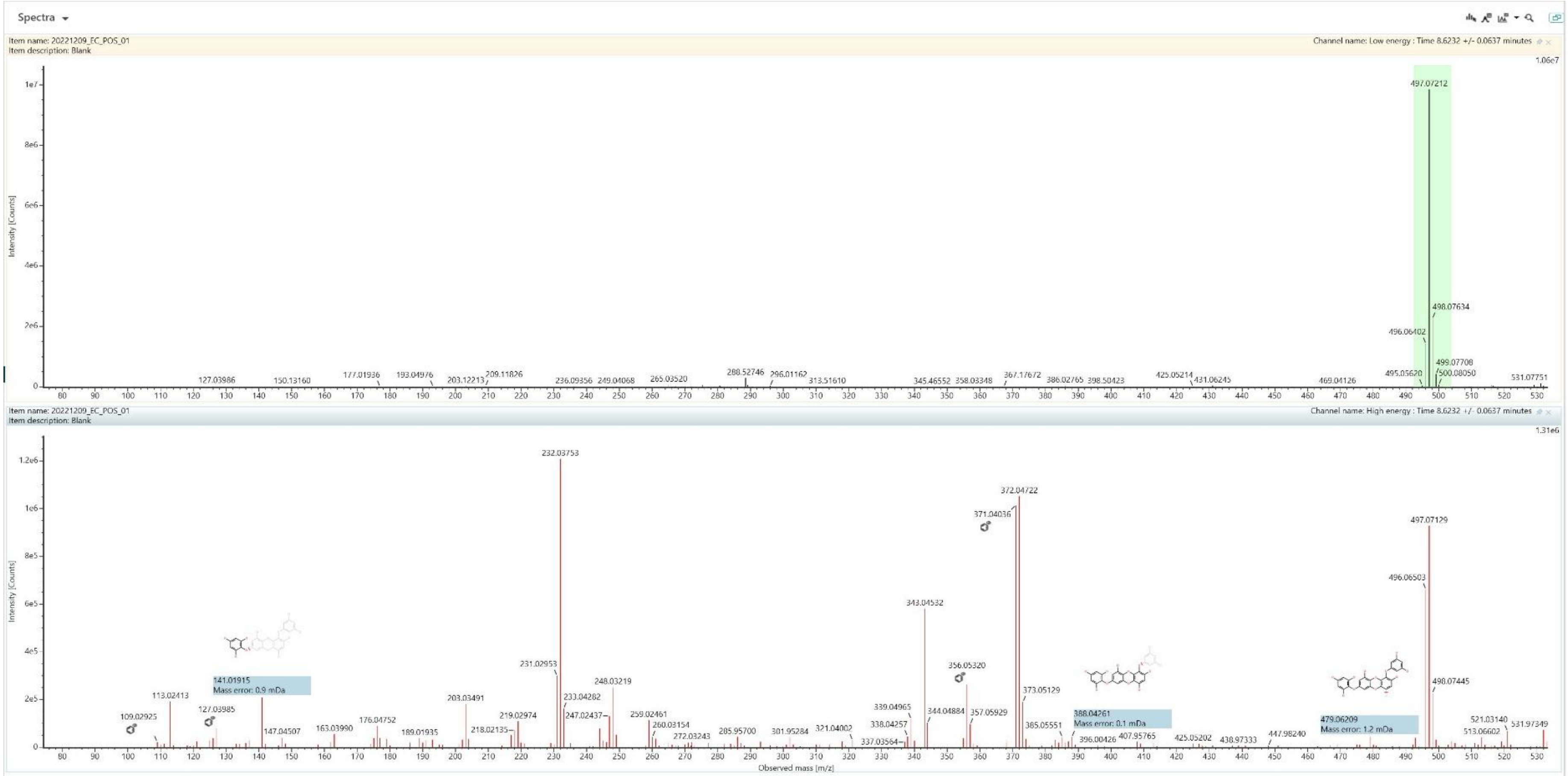

Figure S5. MS/MS spectrum of compound 5 (7-phloroeckol) at RT 8.62 min. Precursor ion $[M+H]^+$ at *m/z* 497.0721.

8,8-Bieckol

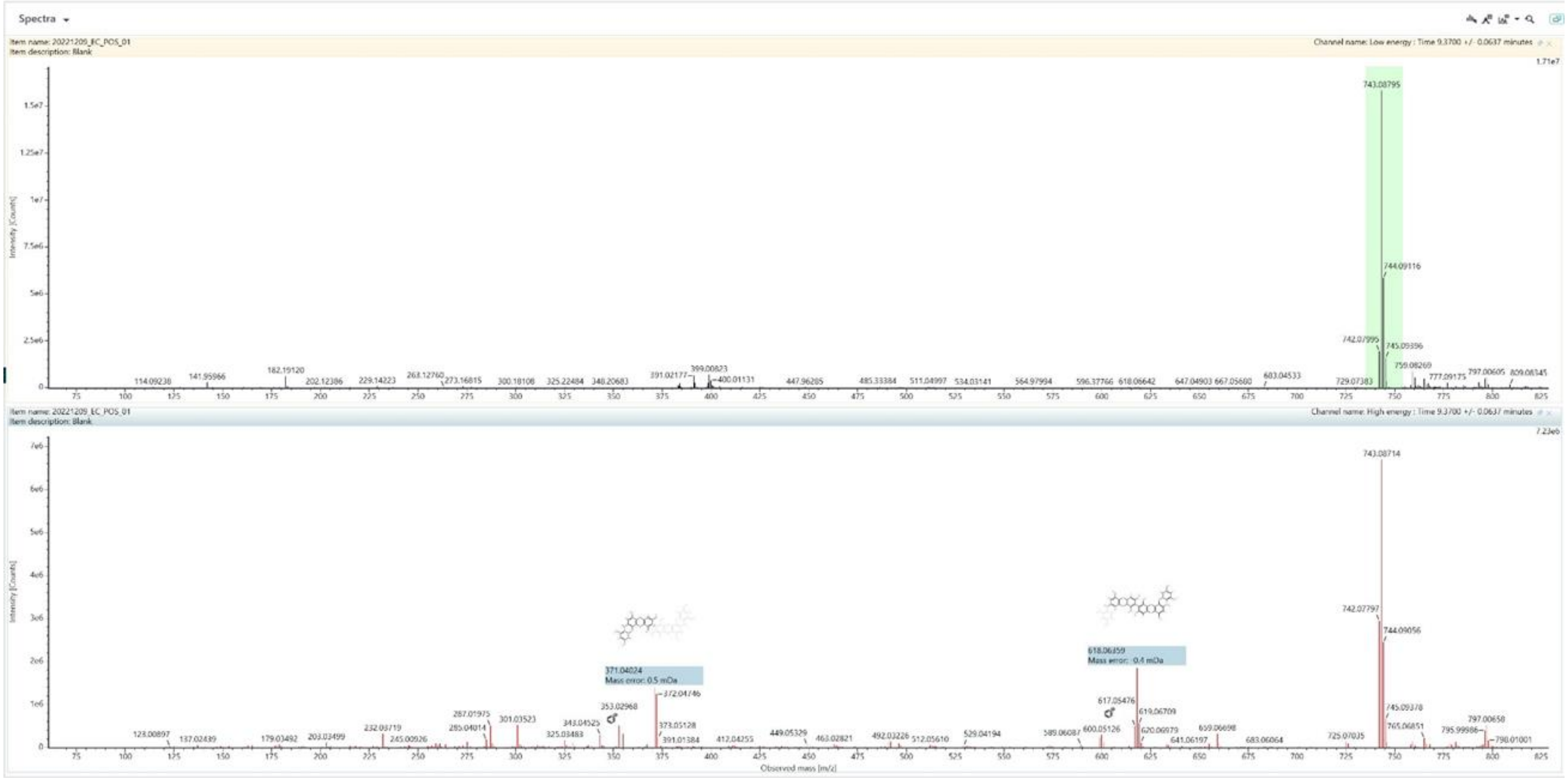

Figure S6. MS/MS spectrum of compound 6 (8,8'-bieckol) at RT 9.37 min. Precursor ion [M+H]+ at *m/z* 743.0879.

2-O-(2,4,6-trihydroxyphenyl)-6,6'-bieckol (isomer)

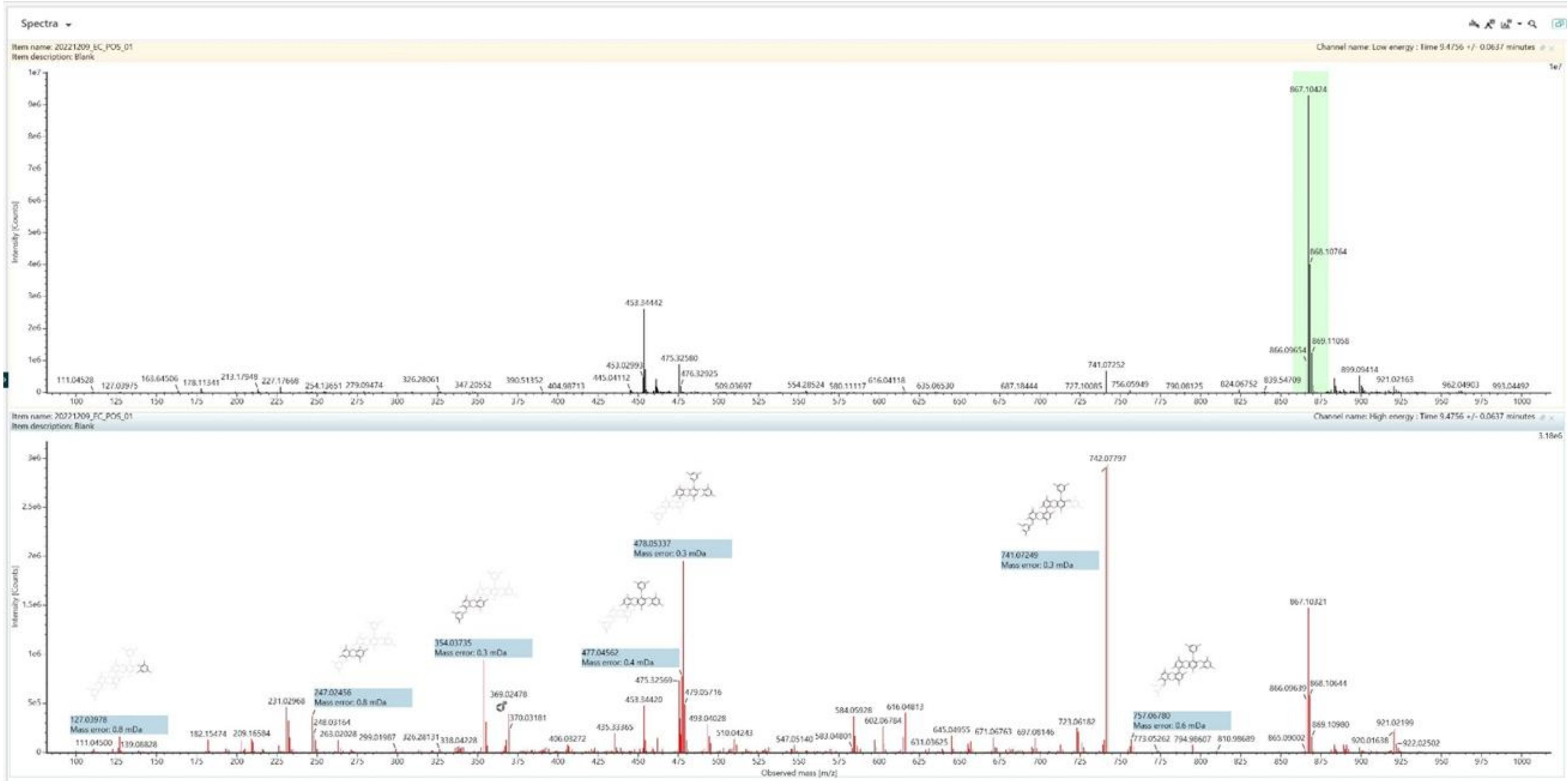

Figure S7. MS/MS spectrum of compound 7 (2-O-(2,4,6-trihydroxyphenyl)-6,6'-bieckol) at RT 9.48 min. Precursor ion $[M+H]^+$ at *m/z* 867.1042.

dibenzodioxin-fucodiphloroethol

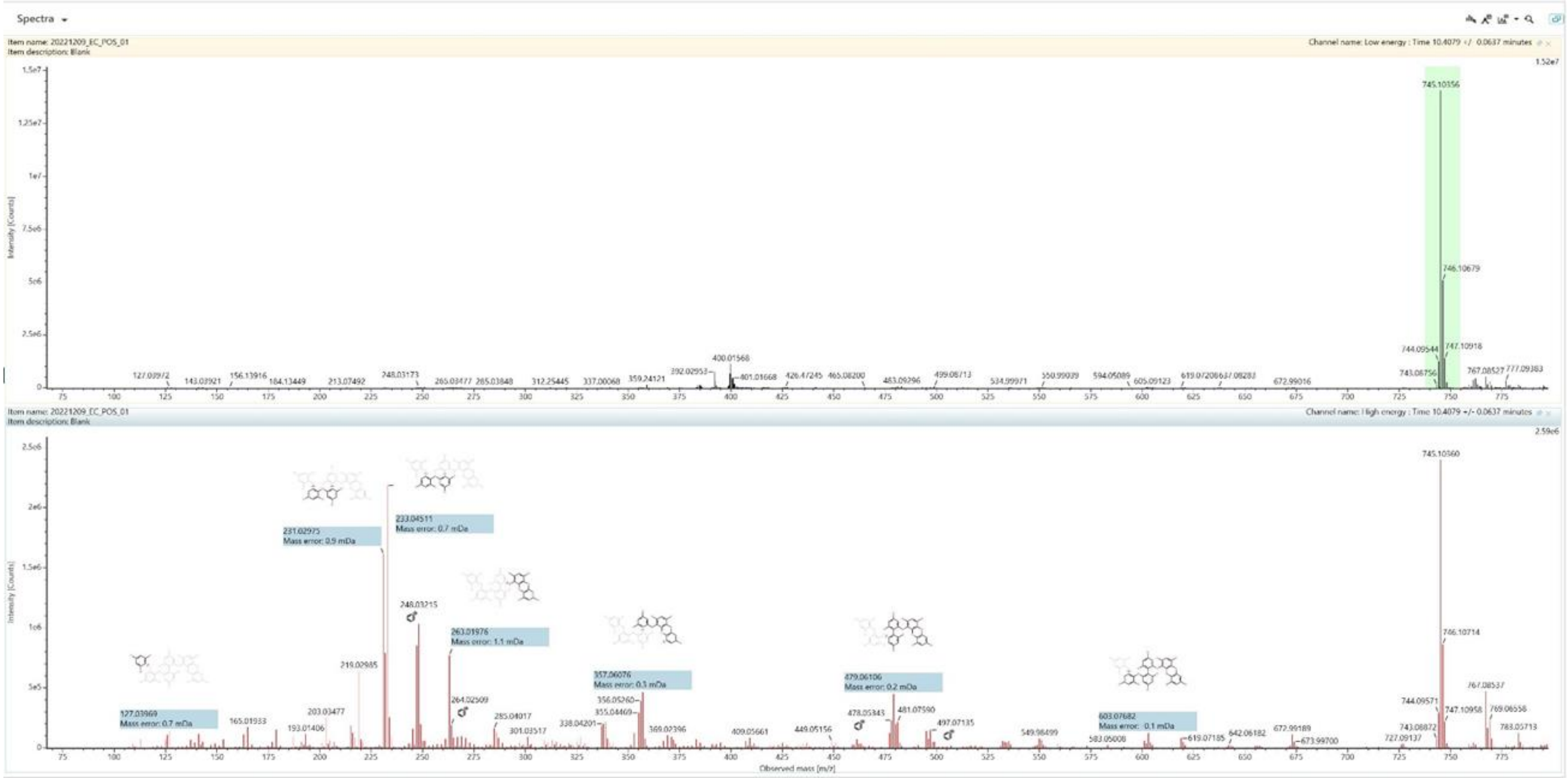

Figure S8. MS/MS spectrum of compound 8 (dibenzodioxin-fucodiphloroethol) at RT 10.41 min. Precursor ion $[M+H]^+$ at *m/z* 745.1036.

Dieckol

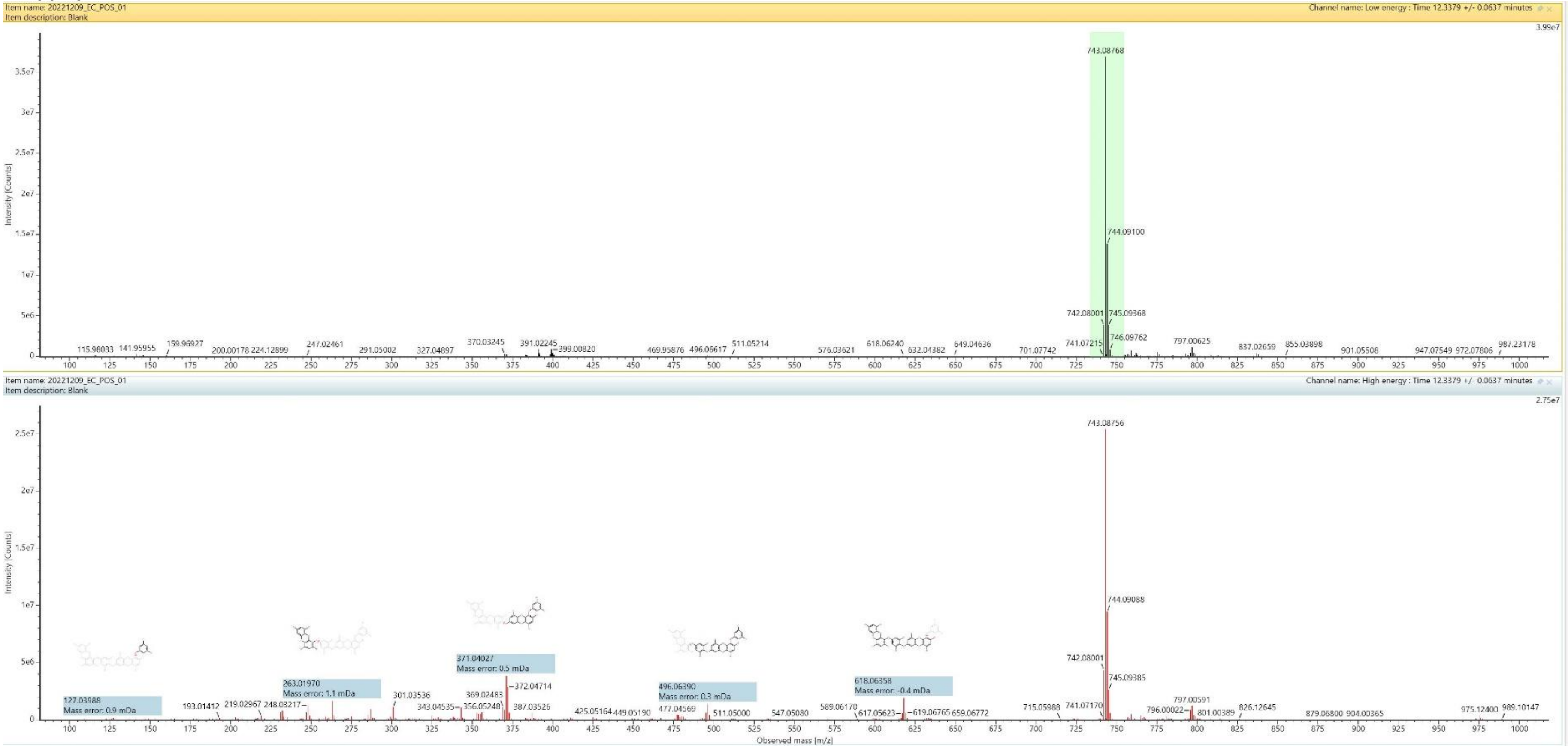

Figure S9. MS/MS spectrum of compound 9 (dieckol) at RT 12.34 min. Precursor ion [M+H]+ at *m/z* 743.0877.

Phlorofucofuroeckol A

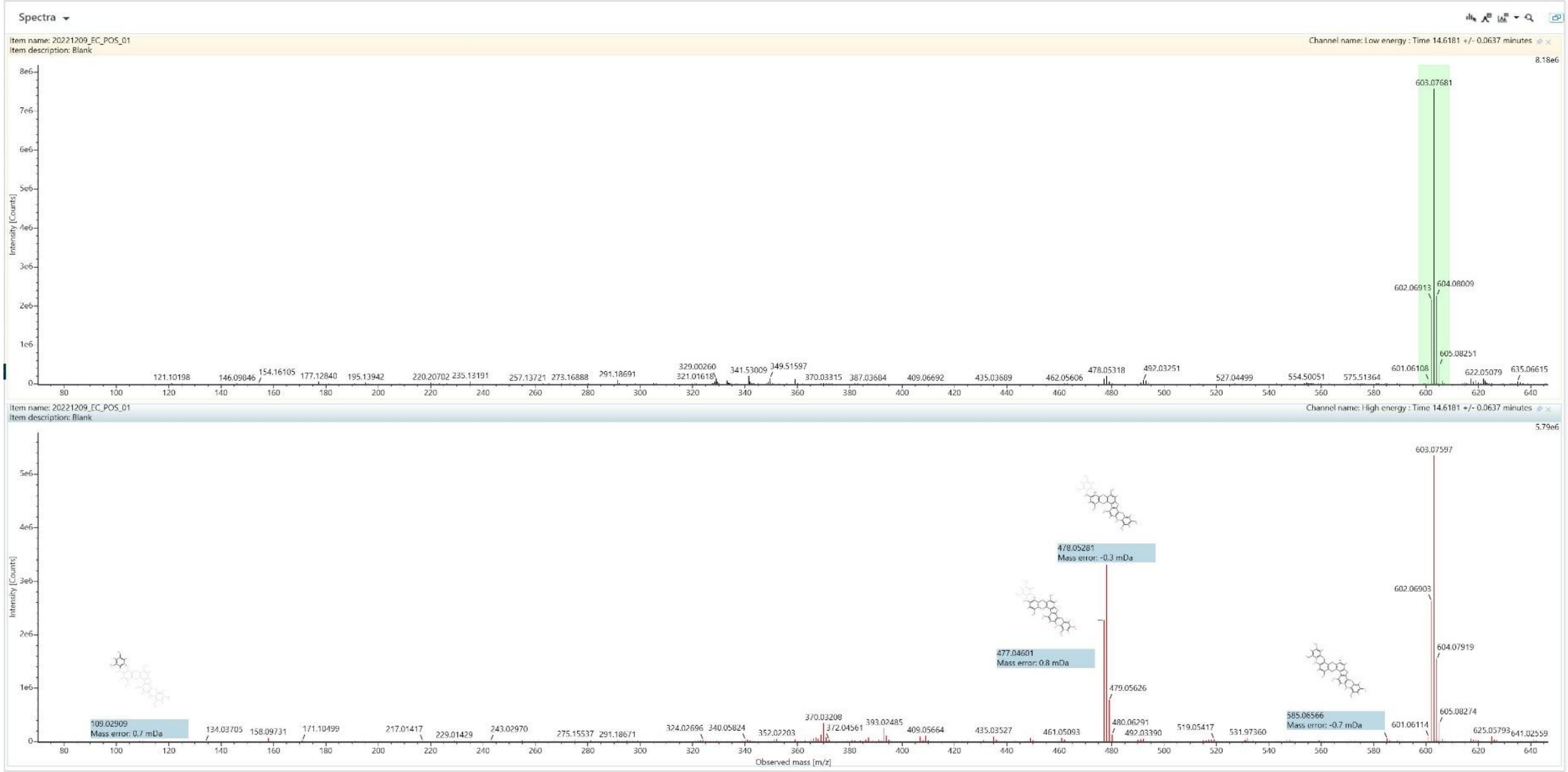

Figure S10. MS/MS spectrum of compound 10 (phlorofucofuroeckol A) at RT 14.62 min. Precursor ion $[M+H]^+$ at *m/z* 603.0768.
